# Patterns, determinants and associated outcomes with adolescent sleep health in Africa: A scoping review

**DOI:** 10.1371/journal.pone.0353671

**Published:** 2026-09-28

**Authors:** Oluwatosin Eunice Olorunmoteni, Biliamin O. Popoola, Jeremiah Oluwatomi I. Daniel, Phelokazi Dlepu, Francesc Xavier Gómez-Olivé, Omobolanle V. Olubowale, Adesegun O. Fatusi, Karine Scheuermaier

**Affiliations:** 1 Department of Paediatrics and Child Health, Faculty of Clinical Sciences, College of Health Sciences Obafemi Awolowo University, Ile-Ife, Osun State, Nigeria; 2 Wits Sleep Laboratory, Brain Function Research Group, Department of Physiology, School of Biomedical Sciences, Faculty of Health Sciences, University of the Witwatersrand, Johannesburg, South Africa; 3 University Library, University of Medical Sciences, Ondo, Ondo State, Nigeria; 4 College of Health Sciences, Obafemi Awolowo University, Ile-Ife, Osun State, Nigeria; 5 MRC/Wits Rural Public Health and Health Transitions Research Unit (Agincourt), School of Public Health, Faculty of Health Sciences, University of the Witwatersrand, Johannesburg, South Africa; 6 School of Public Health, University of Medical Sciences, Ondo, Ondo State, Nigeria; 7 Department of Community Health, College of Health Sciences, Obafemi Awolowo University, Ile-Ife, Osun State, Nigeria; Federal University of Pernambuco: Universidade Federal de Pernambuco, BRAZIL

## Abstract

Sleep health in adolescence is a global public health concern. However, limited studies on adolescent sleep health in Africa exist, and the extent of the available literature on the African continent has not been previously mapped. This review aimed to map the studies on adolescent sleep health in Africa with a focus on the geographical distribution, publication trends, pattern of sleep health measures, prevalence of sleep disorders, the barriers and facilitators to good sleep health, rural-urban disparities, as well as the associations of sleep health with mental health, school schedule and cardio-metabolic risk among the adolescents. All published articles on adolescent sleep health in Africa in English, French, Spanish, Portuguese, and Italian languages were to be included. We conducted a scoping review by searching five databases: MEDLINE (via PubMed), CINAHL (via EBSCOHost), PsycINFO (via EBSCOHost), Scopus, and the African Journal Online (AJOL), from the inception of the databases to January 2025. As previously highlighted in our published protocol, we used the JBI methodology for the scoping review. After screening 921 titles and abstracts and 78 full-text articles, we included 43 articles. There has been an increasing number of studies on adolescent sleep in Africa in the past 25 years. Cross-sectional studies were the most frequent design (67.4%). Most studies focused on sleep quantity and quality, with small sample sizes and scanty evidence on other aspects of sleep health. Inadequate weekday sleep duration was common among adolescents, often compensated by longer weekend sleep durations. Screen use at bedtime, sleeping environment, school schedules, and chronic medical conditions were the major risk factors for sleep disturbances. Poor sleep was associated with poor academic performance, depression, anxiety, and adverse cardiometabolic risk. This review shows a lack of studies on the multidimensional aspects of sleep health and the need for more objective sleep measures to better characterise the sleep health of adolescents in Africa. Larger studies with longitudinal and mixed-method designs are needed to drive the planning and execution of interventions to improve sleep health among African adolescents.

## Introduction

Sleep health is an important concept and a component of health across all ages globally. Understanding sleep health as a multidimensional pattern of sleep-wakefulness tailored to individual, social, and environmental demands is critical in promoting physical and mental well-being. According to Buysse [1], sleep health is not only about the absence of sleep disorders but involves subjective satisfaction, appropriate timing, adequate duration, high efficiency, and sustained alertness during waking hours. Over time, sleep health has evolved from being perceived as a consequence of ill health to a critical determinant of health outcomes, influencing various physical and mental conditions [1–4]. Hence, it is necessary to study sleep health across all ages to ensure the well-being of the entire population.

Research on sleep health has traditionally centred on adult populations [5,6]. The available studies on adolescent sleep health are mainly from high-income countries. Studies from high-income countries (HICs) have robustly linked inadequate sleep with a spectrum of adverse outcomes among adolescents, including physical ailments and behavioural issues [2,7,8]. However, emerging evidence suggests the importance of the multi-dimensions of sleep health among adolescents, revealing that poor sleep health can significantly impact the well-being of this age group, particularly in terms of physical and behavioural health outcomes [4,9]. However, there are limited studies on sleep health among adolescents in low and middle-income countries.

Furthermore, the extent, focus, and geographical distribution of studies on adolescent sleep health, especially within African populations on the African continent, have not been mapped [6,10]. By African, we refer to people residing on the African continent, as this scoping review aims to include all relevant studies from this region. The African continent is a vast and culturally rich landmass with a diversity of cultures, institutions, and ethnicity [11]. The diversity of cultural, ethnic and religious groups on the African continent may affect sleep habits and practices across the continent. Northern African culture/ethnicity and religion are predominantly Arabic and Muslim; Western and Central African ethnicity and religion are African, Muslim and Christian; Eastern African culture includes Ethiopian / Somali / Erythrean and Central African influences, and Muslim and Christian beliefs. Sub-Saharan Africa is a mix of African cultures and European practices, with a predominance of both Christian and Muslim religions. Despite the ethnic, religious, and cultural diversity in Africa, there is a unity in the experience of the continent’s inhabitants that makes this review pivotal for understanding adolescent sleep health across Africa.

To date, the extent and implications of sleep health characteristics such as sleep duration (SD), sleep quality, sleep chronotype (SC), and bedtime variability (BV) among African adolescents have not been comprehensively reviewed. This lack of focused research presents a significant gap, given the potential unique socio-cultural and environmental influences on sleep health within the African context [12–15]. In exploring adolescent sleep health in Africa, it is critical to consider the multiple factors that may be involved, including climatic and genetic factors.

The African climatic zones include the hot and dry arid and semi-arid zones (Sahel region, Kalahari and Namib deserts), the Tropical Savanna grasslands zones (West Sub-saharan Africa and Central Southern Africa), the warm and humid Equatorial (The Congo region and the East African highlands), and the Temperate (Southern Africa) zones [16]. Climatic factors contribute to sleep habits and architecture in Africa, with a higher tendency for afternoon naps and more of the N3 slow-wave sleep architecture among people living in the hot-dry Sahelian conditions than people in temperate and hot-humid climates [17].

Furthermore, genetic factors may contribute to the sleep health of Africans [18]. Though there is a paucity of studies on the association between sleep health and genetic factors among people residing in Africa, findings among individuals of African ancestry in the US showed genetic variations such as Per2, Per3, Clock, and AANAT influencing sleep timing and architecture. These variations predispose these individuals to shorter (<5 hours) or longer sleep durations (> 9 hours) and a higher risk for chronic conditions like cardiovascular diseases [18]. Additionally, obstructive sleep apnea (OSA) has been reported to be more prevalent in individuals of African ancestry and is influenced by genetic factors like the PPARGC1B, HTR2A, and PLEK genes [18].

Moreover, sleep disorders and prevalent diseases such as human African trypanosomiasis, also known as “sleeping sickness,” directly disrupt normal sleep patterns, contributing further to the sleep health burden in the region [19]. Insomnia, which is more commonly reported among African Americans compared to other ethnic groups in the US, has been linked to various social factors, including social class, employment status, education levels, and marital problems, suggesting a complex interplay of environmental and social determinants affecting sleep health [18,19]. The use of khat, specific to Eastern Africa, has been associated with sleep effects [20,21]. Khat (Catha edulis) is a substance of abuse that has been reported to be widely used in East Africa and the Middle East [22,23] It contains the alkaloids, cathinone and cathine, which have similar structural and psychostimulant features to amphetamines [22]. It is often used in combination with other substances, which further worsens its adverse effects on central nervous system function [24]. Higher access to electricity in Africa has been principally in urban areas. With light in the early evenings associated with a delayed circadian phase and bedtimes, adolescents living in urban areas with higher access to electricity may have delayed bedtimes and lower sleep duration [15,25]. Thus, there is an interplay between genetic, physiological, and environmental factors that may affect sleep in Africa, highlighting the complexity of sleep health issues among African adolescents.

Given these complexities, there is an urgent need for a comprehensive review of the available evidence on adolescent sleep health in Africa. Thus, this review aimed to elucidate the determinants and possible outcomes of sleep health among the African adolescent population, focusing on understanding how socio-economic, cultural, environmental, genetic, and mental health/ clinical health-related factors contribute to sleep variability and its associated health implications. It is therefore important to map out studies on adolescent sleep health within the African continent to correctly understand the current situation, identify research gaps, and direct future research. By synthesizing the current literature and identifying research gaps, this scoping review seeks to pave the way for future studies that could inform targeted interventions and public health strategies to improve sleep health and, consequently, the overall well-being of African adolescents. The review, therefore, explored answers to the questions as previously highlighted in the study protocol published in 2023 [26]. Hence, the aim of this review was to describe the evolution of publications in adolescent sleep health in Africa, including the geographical distribution, trends, sources, and types of evidence available on adolescent sleep health in Africa. We also aimed to identify the patterns of sleep characteristics among adolescents in Africa, including investigating rural versus urban differences in sleep health, the barriers and facilitators to good sleep health that have been identified, and the associations between sleep health and mental health, cardiometabolic risk, and school schedule among adolescents in Africa. Furthermore, we reviewed the few interventions that have been implemented for improving the sleep health of adolescents in Africa.

## Methods

### Protocol and registration

The protocol for this scoping review was published in BMJ Open in July 2023, and it contained the list of research questions for this scoping review [26]. The review was conducted using the JBI standards for scoping reviews [27]. As detailed in the proposed review protocol, we deployed the Preferred Reporting Items for Systematic Reviews and Meta-analyses extension for scoping reviews (PRISMA-ScR) to report our findings from the review [28].

### Eligibility criteria

For the review, we included research articles from the global African continent on adolescent sleep characteristics. No time limit was applied in our search for eligible studies for inclusion in the review, to ensure that we capture all relevant perspectives. Studies that report on the sleep health of African adolescents from age 10–19 were included in the review. Studies that primarily focused on children and adolescents were also included if the mean age of the participants was in the adolescent age range, or if a significant proportion of the participants were adolescents, or if the sleep theme being discussed was significant.

As shown in the review protocol, the review plan was to include peer-reviewed studies published in all major languages in Africa, but we found studies published mainly in the English language, with only one in the French language. The French article was translated from French to English by KS. Intentionally, the review excluded information sources from grey literature, such as conference proceedings, web page information, and blogs, to ensure the quality of the source literature. The details of this review’s inclusion and exclusion criteria have been previously published in the protocol [26].

### Information sources and search strategy

We conducted the initial literature search in July 2023 in line with our review objectives. However, the search was repeated in February 2025 to ensure that our review was up-to-date. No changes in thesaurus terms was recorded during the update searches. The databases searched for eligible studies include MEDLINE (via PubMed), CINAHL (via EBSCOHost), PsychINFO (via EBSCOHost), Scopus, and the African Journal Online (AJOL). The search strategies deployed for MEDLINE and Scopus are provided in S1 Table. The strategy was translated for other databases during the search process. References from extracted studies were examined to identify other relevant studies for inclusion in the review. Search results from the databases were deduplicated using the Deduplicator function of Bond University’s Systematic Review Accelerator tool (available at https://sr-accelerator.com/). Fig 1 shows a PRISMA Flow diagram of the evidence search process with 43 studies included in the review.

**Fig 1:**
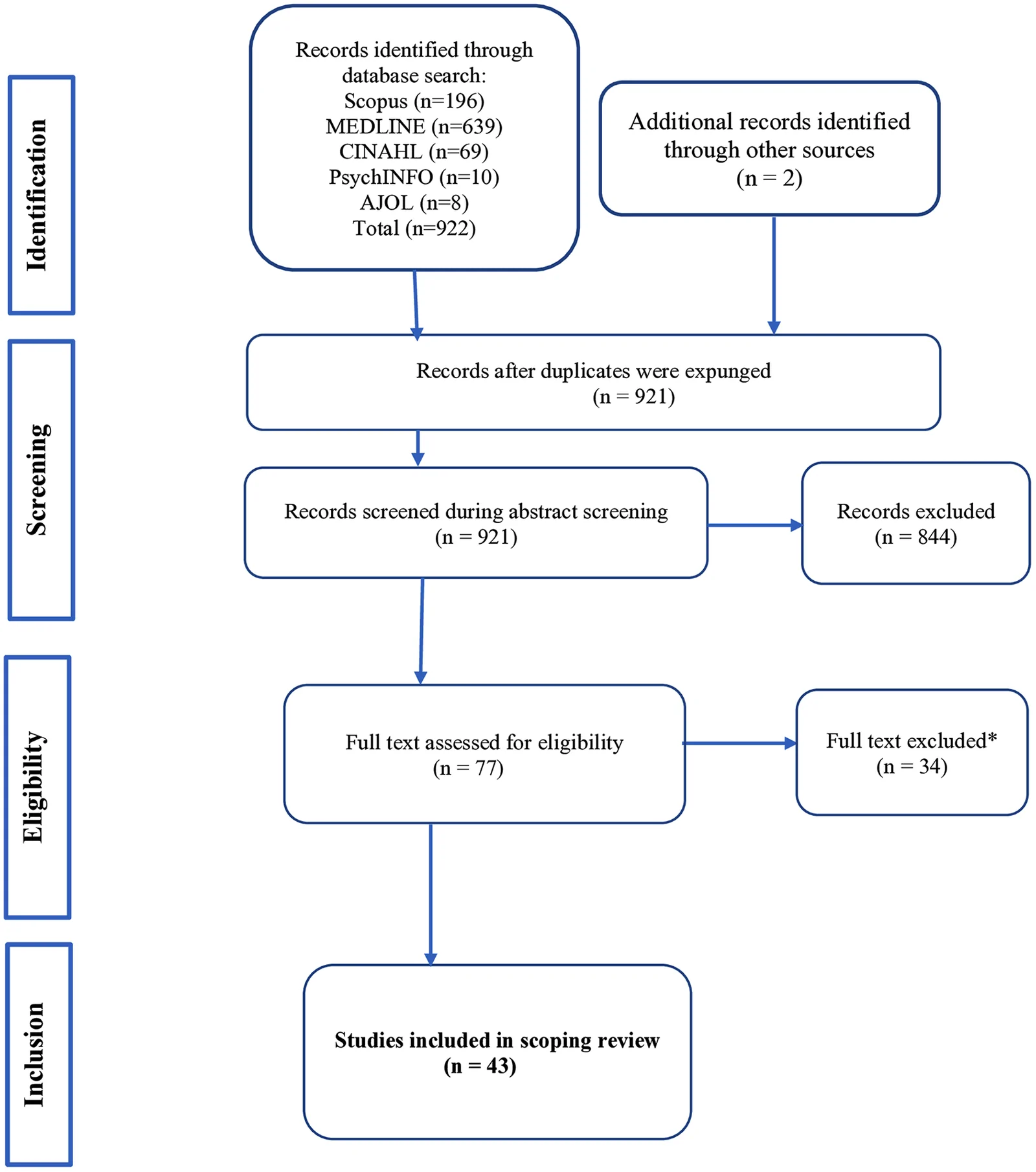
PRISMA Flow diagram indicating evidence search process and number of included studies. *Reasons for exclusion of full texts: No disaggregated data on Adolescent sleep within age 10–19 years = 26; primary focus, not adolescent sleep = 6; full text not retrieved = 2.

### Evidence screening and selection

The evidence screening for the review was conducted using Rayyan (available at https://www.rayyan.ai). Four research team members (OOE, BOP, JOI, and VO) were involved in the abstract and title screening, with Rayyan’s blind function utilized to ensure an independent assessment of the studies. Differences in the screening results were resolved through discussion and consensus after turning off the BLIND function. Furthermore, two reviewers (OOE and JO) independently conducted the full-text screening to ensure stricter consistency and alignment, while BOP acted as an arbitrator to resolve conflicts. Any disagreements over included studies were resolved through discussion and consensus between the reviewers.

### Data extraction

As presented in the review protocol [26], the data extraction tools developed by the researchers were used to extract data from the included studies by three independent reviewers (OOE, PD and JOI). We collated data from the 43 studies that met our study criteria. The analysis and synthesis of the data obtained were guided by our review objectives. The descriptive data obtained included the year of publication, country of publication, study design, and language of publication. We extracted data on the relevant sleep health measures (sleep quality, sleep duration, daytime sleepiness, sleep apnoea), sleep pattern, sleep hygiene and presence of sleep disorders. We also identified the main sleep themes reported in the publications through basic qualitative content analysis. The final results were validated by a librarian (BOP), and a sleep research specialist (KS) as earlier explained in the review protocol [26]. Any disagreement in the extracted data was resolved through discussion and consensus between the reviewers. The findings obtained are presented using charts and tables.

## Results

### Geographical distribution, trends, sources, and types of evidence available on adolescent sleep health in Africa

The descriptive characteristics of the 43 studies included in the review are presented in Table 1. The distribution of the studies across the African regions are also shown on Fig 2. The Northern and Western Africa region had the highest number of studies with 19 and 13 studies from each region, respectively. There were seven studies from the Southern Africa region, three from the Eastern Africa region and none from the Central Africa region. Most of the studies were from Nigeria (10;23.3%) in the Western Africa region, South Africa (7;16.3%) in the Southern Africa region, Tunisia (7;16.3%) and Egypt (7;16.3%) in the Northern Africa region. About three-quarters (29;67.4%) of the studies were cross-sectional in design while prospective longitudinal studies, interventional studies, randomised clinical trials, and case reports contributed 32.6% of the study designs of included studies in the review. The only non-English publication was in French [29]. The trend of evidence on adolescent sleep health in Africa showed a gradual rise in the number of publications evolving from a single publication in the year 2000 to two publications in both the 2001–2005 and the 2006–2010 period with a gradual rise from 2011–2015 to a peak of 22 publications from 2021 to 2025. (Fig 3). Furthermore, the summary of the included studies showed that most studies had a female preponderance (19/43) with five studies having only female participants (Table 1). There was a case report on Kleine-Levin syndrome in a South African adolescent and another case report on Narcolepsy in a Nigerian adolescent. The cross-sectional studies had numbers of participants ranging from 20 to 1695, while the longitudinal studies had participants between 23 and 3273. The majority of the studies utilised questionnaires to obtain information on the adolescents’ sleep health, six studies utilised polysomnography, while two studies used accelerometers, two used actigraphy and one utilized a wearable device. The Pittsburgh Sleep Quality Index was the most commonly used validated sleep questionnaire (14/36; 38.9%). However, many studies used a few questions about duration or quality of sleep to determine adolescents’ bedtime, waketime, and sleep hygiene. The types of sleep health measures reported in the publications and the various outcome measures studied are summarised in Table 2.

**Table 1:** Descriptive characteristics of included publications.

Characteristics	Frequency (n)	Percentage (%)
Country of Publication		
Nigeria	9	23.3
South Africa	7	16.3
Egypt	7	16.3
Tunisia	7	16.3
Others*	12	27.8
Study Design		
Cross-sectional	29	67.4
Longitudinal (cohort/ case-control)	6	14.0
RCT#	4	9.2
Interventional	2	4.7
Case report	2	4.7
Language of Publication		
English	42	97.7
French	1	2.3

*Morocco had four publications, Senegal and Uganda had two publications each; Sudan, Cote d’Ivoire and Ethiopia had one publication each while a single publication was conducted in Cape Verde and Mozambique.

# RCT: Randomised Clinical Trial

**Table 2:** Findings on prevalence and pattern of sleep characteristics among African adolescents, in chronological order.

Reference	Year	Study design, Sample size, Participants’ characteristics	Sleep health variables	Outcome domain studied	Sleep measure	Patterns of sleep characteristics
	**Country**	**(Age, Sex)**				
Galal *et al* [30]	2001 Egypt	Cross-sectional n: 863 age: 12–18 y sex: 54% M	Sleep disturbance	Sleep perceptions, physical health symptoms	**Questionnaire** used was an adaptation of the ‘Health of Youth Questionnaire’. Self-reported Sleep disturbance was assessed by adolescents on how frequently they experienced disturbed `sleep in the preceding week. Perceptions of health and sleep were rated as very healthy, healthy, average or bad.	Significantly, more boys than girls perceived their sleep health as very healthy
Reid *et al* [31]	2002 South Africa	Cross-sectional n: 825 age: 16 ± 1 sex: 61% F	Sleep onset latency, Sleep duration, Sleep quality	Sleep hygiene and habits	**Questionnaire** included questions used at the University of Witwatersrand Sleep Laboratory: - Sleep habits: times they go to bed and wake up, how long they take to fall asleep (subjective sleep onset latency; SOLs), how many times they had woken up and for how long they remain awake (wake after sleep onset; WASO) on the previous night, use of medications to sleep and alarm clock to wake up. - Daytime functioning the previous day. They were asked if they took a nap, the nap duration if they exercised, and for how long, if they took caffeinated beverages and how many cups. - Subjective morning alertness on the day of filling out the questionnaire. They answered whether or not they thought they could fall asleep within five minutes if they had the chance.	Frequency of awakening: at least once/night: 52%; WASO Duration of awakening ≤ 30 minutes: 6% Mean SOL: 17 minutes (with 45% SOL < 10 minutes) 72% consumed caffeinated beverages the day before the survey 8% took sleep medications 62% needed an alarm or wake-up call (external aids) in the morning.
Bénéfice *et al* [32]	2004 Senegal	Longitudinal (three consecutive years), Cohort n: 40 age: 13.3 ± 0.5 at baseline sex: 100% F	Sleep duration Sleep quality Sleep variability	Nutritional status; Growth Puberty Season	Sleep duration: **Accelerometry** counts of smooth movements between 20:00h and 8:00h were used to determine sleep length. Sleep onset: is determined as a consecutive sequence of zero counts and wake-time as successive counts above 100, Sleep quality: based on the depth of sleep derived from mean counts of movements/minute during the sleep period. Sleep variability: based on standard deviation in mean counts during sleep period.	Mean sleep duration: 8.5 ± 0.9 Sleep duration improved by 30 minutes between the baseline and final measurements. Sleep quality improved as the number of movements during sleep reduced progressively over the three years
Garnier & Bénéfice [33]	2006 Senegal	Cross-sectional n: 80 age: 14.2 ± 0.6 for boys and 14.4 ± 0.5 for girls sex: 50.0% F	Sleep duration, Sleep efficiency, Sleep onset latency.	Sleep and Physical activity	**Acceleromete**r: Used an Excel Visual Basics macro programming (Microsoft Corporation, Redmond, WA) to objectively calculate characteristics of sleep and diurnal physical inactivity derived from the movement registration recorded by the accelerometer.	Sex differences in sleep and physical activity patterns: - Girls: mean sleep duration of 8 hours and 45 minutes; longer than boys (p < 0.05). - Sleep efficiency: 91.6%; Inactive for 4 hours and 23 minutes/day? - Boys: mean sleep duration of 8 hours and 9 minutes; Lower sleep efficiency than girls: (88.3%); were more active than girls with only 2 hours and 49 minutes of inactivity.
Cockcroft *et al* [34]	2009; South Africa	Case-control n: 23 children with ADHD −12 medicated −11 un-medicated age:6.4–12.7; sex: 69.6% F;	Daytime sleepiness, Sleep disorders.	Effect of methylphenidate on Perceived Levels of daytime sleepiness and prevalence of sleep disorders in medicated versus unmedicated Children with ADHD in the afternoon (13:00–15:00).	**Questionnaires** (and a Parental Questionnaire on Sleep Habits). Wits Faces Sleepiness Scale: filled by children to measure their daytime sleepiness at 8:30 am, (before any break time), and at 13h00(after the lunch break) on the same day Parental Questionnaire on Sleep Habits: 12 open- ended questions, developed by the researchers. Measured total sleep time, sleep latency, nighttime arousals, daytime naps, symptoms and signs of the common sleep disorders according to DSM-IV and a parent-reported visual analogue scale children’s general levels of sleepiness rated as “not at all sleepy” to “very sleepy” between 3:00 and 15:00 hours and between 17:00 and 19:00 the hours	No significant difference in the overall prevalence of disordered sleep symptoms between medicated and unmedicated children with ADHD (p = 0.06) but a high prevalence of leg movements (50% vs36%) and nightmares (46% vs36%) in both groups Snoring (16%), apnea/ gasping (8%), restlessness (8%), various sleep positions (8%) and bedwetting (16%) were only reported among the medicated children. Between 08:30 and 13:00Perceived daytime sleepiness increased significantly in the medicated children (t = −2.59; df = 8; p < 0.05), compared to the un-medicated group. Parents of the medicated children perceived significantly increasing sleepiness in their children as the day progressed, compared to the parents of the unmedicated children (t = 2.45; df = 8; p < 0.05)
Aalouane *et al* [29]	2011; Morocco	Cross-sectional n: 1451 sex: 56.7% M	Sleep pattern	School performance	Questionnaires (Self-report questionnaire containing questions on bedtime, waketime, sleepiness during classroom and school performance over the past three months).	Twenty-two per cent of the girls and 18.1% of the boys had symptoms of disordered sleep. Daytime somnolence in the classroom was found in 17.6% of the girls and 12.8% of the boys.
Fakier & Wild [35]	2011 South Africa	Cross-sectional of adolescents with and without learning difficulties (LD) n: 703 LD:309 (44%); Control: 394 age: LD:13–20 Control: 14–18 sex: LD: 66% M Non-LD: 47% M	Sleep pattern	Learning difficulties and substance use	Questionnaires: assessed Sleep problems using the sum of four items from the Child Behaviour Checklist on trouble falling asleep, trouble staying asleep, feeling tired in the morning and having a problem with sleepiness during the day)	No data on specific sleep characteristics but reported associations between sleep. problems, learning difficulties (LD) and substance use. Controlling for effect size, adolescents with sleep problems were more likely to use cannabis and inhalants.
Alabi *et al* [36]	2012 Nigeria	Cross-sectional n: 909 age: 3–16 sex: 50.0%F	Snoring, Sleep Disordered Breathing;	Snoring symptoms and complications	Questionnaires (Parent-report questionnaire on sleep habits and snoring)	34.2% of snorers with various sleep-related issues. 34.1% snorers with restlessness during sleep. 39.5% snorers with cessation of breathing. Obesity: present in 33.5% of snorers, and 17.5% showed signs of adenotonsillar hypertrophy.
Borchers & Randler [37]	2012 Côte d’Ivoire	Cross-sectional; n: 588; age: 10–15 (12.72 ± 1.63) sex: 57.5% F	Sleep duration, Sleep-wake cycle, Sleep regularity	Age, gender, religion, occupation	Questionnaires. The questions asked are as follows: “Think of one typical day during the week (when you are working or at school): - When do you get up in the morning? - When do you go to bed in the evening? Think about a day at the weekend (when you do not work): - When do you get up in the morning? - When do you go to bed in the evening? - Do you sleep/ nap during the day? If yes, how often do you sleep during the day? How many hours every time (nap)?” Using bed and rise times, they calculated a proxy of sleep duration (time spent in bed on weekdays and weekends) and a proxy of a chronotype measurement (midpoint of sleep on weekdays and weekends). To calculate the mid-sleep time they used the midpoint of time in bed (midpoint between bedtime and rise time).	The mean midpoint of sleep on weekends (1:37 ± 00:42) was significantly later than on weekdays (1:26 ± 00:30) with p < 0.001. Adolescents had later bedtimes, later waketimes, shorter sleep duration and later midpoint of sleep on weekends than weekdays (all p < 0.001).
Maduabuchi *et al* [13]	2014 Nigeria	Cross-sectional; n: 443 age: 13–19 sex: 59.4% F	Symptoms of disordered sleep, Sleep duration on weekdays and weekends, Sleep quality, Daytime sleepiness, Sleep onset latency	Sleep hygiene	Questionnaires (PSQI, ESS)	The mean sleep duration during weekdays (7.84 ± 1.9 hours) was significantly shorter than on weekends (8.65 ± 2.07 hours) (p < 0.001). 22.8% (101/443) had abnormal sleep onset latency. A high chance of sleeping was reported while sitting and reading in 14.4% (64/443), while sitting and talking to someone (7.4%) and while driving (2.7%).
Lachman [38]	2014 South Africa	Case report n (age): 1 (14y)	Hypersomnia	Not applicable	Case report	First documented case of Kleine-Levin syndrome (KLS) in South Africa; associated with hypersomnia Diagnostic challenges were highlighted, including misdiagnosis as bipolar disorder or seizure disorder, due to the rarity and overlapping symptoms of KLS with other neuropsychiatric conditions.
Sanya *et al* [39]	2015 Nigeria	Cross-sectional n: 1033 age: 12–19 (15.3 ± 1.6) sex: 51.7% F	Sleep pattern, Sleep duration, Daytime sleepiness	Sleep hygiene	Questionnaires (Sleep habits) The authors designed a sleep questionnaire from previous studies. It contained questions on “total duration of sleep in a day; time for bed; sleep latency; nocturnal awakenings (weekly and daily frequency, duration, reasons); waketime, time to leave the bed and daytime (school time) napping habits. The duration of the nighttime sleep was calculated as the difference between bedtime and waketime”	Adolescents had significantly shorter sleep duration on weekdays (9.33 ± 2.29 h) compared to 10.09 ± 1.32 h on weekends (p < 0.05) 13.3% of the adolescents had insufficient sleep duration (<8 h) 41% had adequate (>9 h) SD while 44%borderline SD (8–9 h) Daytime sleepiness was reported in 8.2%
Balogun *et al* [12]	2017 Nigeria	Cross-sectional n: 450 age: 10–18(13.4 ± 1.2) sex: 52% F	Sleep quality, Sleep quality, Sleep onset latency	Sleep hygiene, Symptoms of disordered sleep	Questionnaire (PSQI)	Poor sleep quality: 50.2% Prevalent frequent awakenings (23.3%), and reliance on sleep medication (28.7%)
Christoph *et al* [40]	2017 Uganda	Cross-sectional n: 148 age: 11–16 (12.1 ± 1.1) sex: 61.8% M	Sleep disorders, Sleep quality	BMI, Physical activity	Questionnaires (Four questions) • “During the past week, what time have you usually turned out the light and gone to sleep on school days/weekend days? • During the past week, what time do you usually wake up in the morning on school days/weekend days? • During the past week, how would you rate your sleep quantity? • During the past week, how well did you sleep? (subjective sleep quality”	Mean self-reported sleep duration Rural: 8.5 ± 1.1 h Urban: 8.4 ± 1.3 h No significant difference in sleep duration between rural and urban adolescents Self-rated sleep quantity as very good: Rural: 86.7% Urban: 87.7% Self-rated sleep quality as very good: Rural: 96.0% Urban: 89.0%
Peter *et al* [41]	2017 Nigeria	Cross-sectional n: 353 age: 15.89 ± 1.93 sex: 61.8% M	Sleep problem, Sleep disorder, Sleep duration, Daytime Sleepiness, Sleep Regularity	Sleep hygiene	Questionnaire (BEARS Sleep Screening Tool): • **B**edtime problems • **E**xcessive daytime sleepiness • **A**wakenings during the night • **R**egularity and duration of sleep **S**leep-disordered breathing	Frequent awakenings 34.6%; Problem falling back asleep after an awakening: 20.1% Excessive daytime sleepiness: 20.9%; Sleep-disordered breathing: 4.8% Difficulties with bedtime routines: 15.3% Mean sleep duration Early adolescents: 7.19 ± 1.26h Middle adolescents:7.13 ± 1.13 Late adolescents:7.16 ± 1.28 h
Regaieg *et al* [42]	2017 Tunisia	Cross-sectional n: 1695 age: 15–18 years sex: 56.3% F	Sleep duration	Obesity (BMI and WC)	Questionnaire (Self-report of a typical sleep duration on school and weekend nights. The average time spent sleeping per day was calculated for each participant as [(minutes on weekdays x 5) + (minutes on weekends x 2)]/7. Short sleep duration was defined as <8 hours.	71.1% of adolescents had short sleep duration The prevalence of obesity and the waist circumference value was significantly higher for short sleepers as compared with normal sleepers (p = 0.04 and p = 0.05) respectively).
Cheikh *et al* [43]	2018 Tunisia	Randomised (double-blind design) clinical trial n: 10 Teenage athletes; age: 15.4 ± 0.3 sex: 100.0% M	Sleep architecture	Physical and cognitive wellness following melatonin administration (MEL)	Polysomnography	Following MEL, total sleep time increased by 24.55 min, Sleep efficiency by 4.47% (p < 0.001), and SOL by 8.45 min while Nighttime awakenings reduced (18.95 min versus 29.95 min; p < 0.001), N3-sleep increased (19.86% versus 18.3%; p < 0.05) and REM-sleep increased (18.72% versus 16.57%; p < 0.00) compared to the placebo condition
Olorunmoteni *et al* [15]	2018 Nigeria	Cross-sectional n: 346 age: 10–19 (13.5 ± 2.29) sex: 51.4% F	Sleep pattern	Socio-environmental factors, electronic device use	Questionnaire (Adolescent Sleep Habits Questionnaire)	Insufficient sleep: weekdays = 44.4%, weekends = 5% Mean total sleep duration on weekdays (7 h 15 min ± 86 min) was significantly lower than at weekends (9h 08 min ± 113 min) (p < 0.001) Lower social class and public school attendance are associated with longer weekday sleep duration; Female gender, younger age, and living in a polygamous family are linked to longer sleep on weekends.
Carneiro *et al* [44]	2019 Cape Verde (CV) & Mozambique (M)	Cross-sectional n: 660 CV: n: 206 M: 445 age: 2 -15y &4-13y 2 – 7y: CV: 61%; M: 47% 8 – 10y: CV: 24%; M: 40% 11–15 CV: 15%; M: 13% sex: 51% F	Sleep disorder	Sleep hygiene	Questionnaires (CSHQ)	**CV sample:** 29.9% with sleep disorders; restless sleep (67%); need awakening by others (46%); falling asleep in another bed (63%): Usually or sometimes used television as a sleep aid (30%); needs an object to fall asleep (12%). **M sample:** 28.4% with sleep disorders. Restless sleep (41%). Need awakening by others (85%); falling asleep in another bed (29%); Usually or sometimes used television as a sleep aid (33%); needs an object to fall asleep (16%) More educated parents had children with better sleep patterns. In both populations, the median CSHQ score was not statistically different between the age categories. Among those in the 11–15 years old group, For CV: mean sleep duration on weekdays:11.70 ± 1.87 h while M: mean sleep duration on weekdays: 9.13 ± 1.04 h Bedtimes are similar in both samples of adolescents the CV sample had a later wake time of 7.19 ± 1.87 am than the M sample of 5.43 ± 0.27 Those average sleep durations included nap and WE sleep.
Hanson et al [45]	2019 South Africa	Longitudinal n: 1414 age: 12–17 sex: 52.7% F Group-based multi-trajectory modelling was done to group the participants into classes based on longitudinal trajectories (time spent in physical activity/ sports, sedentary behaviour, and sleep on weekdays and weekends	Sleep pattern	Sedentary lifestyle, physical activity	Questionnaires (Few questions): clock times at which adolescents went to bed and woke up on school nights and weekends/holidays, then calculated hours of sleep.	Maternal education is significantly associated with sleep trajectories (Adolescents with mothers with secondary school education are more likely to be in the 7h or 8h per night sleep trajectory compared to the 9h per night trajectory.) Sedentary behaviour and sleep trajectories did not cluster together with (were independent of) the physical activity domains
El-Baroudy *et al* [46]	2020 Egypt	Cross-sectional n: 80 (40 children on regular hemodialysis, 40 controls) age: 6–15 sex: Patients: 65% F Controls: 55% F	Sleep quality Daytime sleepiness Sleep pattern	Kidney dysfunction	Questionnaires (modified ESS) and Polysomnography	Children with end-stage renal disease had: -Lower sleep efficiency in (92.84 ± 6.62) compared to controls (96.12 ± 3.60) (p = 0.007) -Shorter sleep duration in minutes (358.2 ± 40.8) than controls (438.5 ± 42.7) (p < 0.001) - Higher AHI (4.8 ± 3.0) than controls (2.7 ± 2.1) (p = 0.004) - Higher periodic limb movement index -Higher Excessive daytime sleepiness (15%) than controls(2.5%)
Abdelmaksoud *et al* [47]	2021 Egypt	Case-control n: 120 (60 Type 1 Diabetes mellitus, 60 controls); age: 10–18 (13.57 ± 1.76); sex: TID: 30% F; Controls: 36% F	Sleep quality, Daytime sleepiness, Sleep architecture	Glycemic control, insulin resistance and microvascular complications in Type 1 Diabetes Mellitus (TID)	Questionnaire (ESS-CHAD) and Polysomnography	Adolescents with T1D had significantly lower ESS-CHAD (P < 0.001) than controls. Adolescents with T1D had significantly lower sleep efficiency and REM sleep with higher SOL and AHI (all *p* < 0.001) compared to controls. T1D + microvascular complications associated with lower sleep efficiency and less REM sleep. Insulin sensitivity positively correlated with sleep efficiency; and negatively correlated with periodic limb movement index (PLMI).
Micklesfield *et al* [48]	2021 South Africa	Longitudinal (Birth-to-Twenty Plus) cohort Longitudinal association between school night sleep between ages of 12–18 and anthropometric measurements at 18 years old (adjusting for physical activity and sedentarity) Inclusion criteria: at least two data points between 12 and 18 years of age for physical activity, sedentary behavior and sleep and have either anthropometric or body composition data at age 18 years. n: 1337 age: 12–18; sex: 52% F	Sleep duration	Body composition outcomes, Physical activity, Sedentary lifestyle	Questionnaire (sleep duration calculated from self-reported school night bedtime and waketime)	Sex differences in sleep patterns: - Females: 65% slept 8 hours or less on school nights - Males: 35% slept 8 hours of less on school nights
El Refay *et al* [49]	2021 Egypt	Cross-sectional n: 765 age: 4–16 sex: 53.3% M	Sleep disturbance	Psychological stress, Anxiety symptoms	Questionnaire (SDSC)	Overall mean Sleep Disturbance Scale for Children score (SDSC): 44.6 ± 11.72. Symptoms suggestive of sleep disturbance 65.6%. Disorders of initiating and maintaining sleep: 168 (33.4%) Excessive somnolence: 79 (15.7%). Prevalence of Anxiety: 48%. A significant positive correlation was found between family income affection, SDSC, and anxiety (p = 0.001 and 0.004).
Wolde *et al* [20]	2022 Ethiopia	Cross-sectional n: 799; age: 10–19 sex: 52.1% M	Sleep quality	Inhalant, khat, and internet use disorders; Physical illness	Questionnaire (PSQI)	38% had poor sleep quality (PSQ). Adolescents with physical illness had about 4 times higher odds of association with PSQ (AOR = 3.76 and inhalant use disorder (AOR = 1.98), internet addiction (AOR = 3.33), and khat use disorder (AOR = 1.74), were positively associated with PSQ.
Moustakbal & Maataoui [50]	2022 Morocco	Cross-sectional n: 730; age: 15.64 ± 1.66 sex: 55.5% F	Sleep duration, Sleep quality, Sleep onset latency	Mobile phone use	Questionnaire (PSQI)	Mean total sleep time = 8.01 hours. Poor sleep quality: 72% More in older adolescents, those from rural areas, and those with physical or psychological health issues.
Elfaki *et al* [51]	2023 Sudan	Cross-sectional n: 921; age: 10–15 sex: 57.9% F	Sleep duration	Metabolic syndrome	Questionnaire: Sleep duration was derived by asking how long on average the adolescents slept during the night in the past six months. For adolescents aged <14 years, short sleep is defined as <9 h and normal sleep as 9–11 h. For adolescents aged 14–15 years, short sleep is < 8 h and normal sleep is 8–10 h.	Prevalence of short sleep was 55.0%. Males: 53.1% Females: 56.5%
Guembri [52]	2023 Tunisia	Cross-sectional n: 72; age: U14:13.44 ± 0.5 sex: 100.0% F	Sleep quality, Daytime sleepiness, Sleep disorders	Sleep hygiene, balance, strength, agility, and maximum aerobic speed	Questionnaires (PSQI, ISI, and ESS)	Poor sleep quality: • 61.1% for the U14 group • 63.8% for the U17 group Mild insomnia: 8.3% of U14 players and 8.4% of U17 players Excessive daytime sleepiness: • 19.4% of the U14 group • 22.3% of the U17 group
Mlouki *et al* [53]	2023 Tunisia	Cross-sectional n: 404; age: 16–18 (17.18 ± 1.5) sex: 63.0% F	Daytime sleepiness, Sleep quality	Adverse childhood experience	Questionnaire (PSQI)	A high prevalence of sleep disturbance which is defined as PSQI>5: 94%. Prevalence of internet addiction: 80.2%.
Olorunmoteni *et al* [54]	2023 Nigeria	Cross-sectional n: 448; age: 10–19; sex: 57.8% F	Sleep quality	School schedule, Mental Health	Questionnaire (PSQI)	High prevalence of poor sleep quality: 85% (particularly in older adolescents). Insufficient sleep: 55.1% on weekdays, 34.8% on weekends.
Tantawy *et al* [55]	2023 Egypt	Cross-sectional n: 20; age: 6–18 (10.22 ± 3.28) sex: 63.3% M	Sleep disordered breathing (OSA)	Pulmonary function in sickle cell anaemia (SCA)	Questionnaire (modified STOPBANG) and Polysomnography	60% of participants had Obstructive Sleep Apnea (OSA) Six children (20%) had a high risk for obstructive sleep apnea (OSA), while nine (30%) were at intermediate risk. Children with moderate to severe OSA had significantly higher tricuspid regurgitation, velocity (p = 0.007) and left middle cerebral artery flow velocity(higher stroke risk) (p = 0.049) when compared to those with AHI < 5.
El‑Jaziz *et al* [56]	2023 Morocco	Prospective cohort with repeated measurements N: 50 Age: 17.22 ± 1.15 y Sex: 66% F	Sleep quality, chronotype, TIB, sleep latency, WASO, total sleep time, sleep efficiency derived from the questionnaires.	Aerobic performance during Ramadan fasting	Questionnaires (Morningness‑Eveningness questionnaire, Sleep Timing Questionnaire, PSQI)	Fasting during Ramadan was significantly associated with reduced sleep quality and aerobic performance; Being a morning type was associated with higher TIB only. Chronotype was not associated with aerobic performance. There was no effect of Ramadan fasting on sleep efficiency, sleep latency, or WASO.
Olorunmoteni *et al* [57]	2024 Nigeria	Cross-sectional; N:900 Age: 13–19 59% female	Sleep health (sleep quality, duration, daytime sleepiness, sleep apnea)	Multi-dimensions of sleep health; Rural vs Urban residence	Questionnaires: Short sleep (<8.5 hrs), PSQI, ESS‑CHAD, Teen STOP‑BANG	79% had short sleep; 14.9% had poor sleep quality; 9.6% had excessive daytime sleepiness; 9.8% had sleep apnea risk; urban adolescents showed significantly higher prevalence of those markers of poor sleep health (p values: .004, < .001, .044, .006)
El Mlili *et al* [58]	2024 Morocco	Cross-sectional; n:60 age: 6–17 y girls: 75%	Sleep quality	Sleep quality in children and adolescents with Type I DM	Questionnaire: PSQI	Poor sleep quality: 44.4% girls, 33.3% boys in Type I DM.
Saifeldin *et al* [59]	2024 Egypt	Intervention study: Twin Block insertion for mandibular malocclusion. n: 24 age:11–13 y sex: 100% F Exclusion criteria: diagnosed sleep apnoea or adenotonsillar hypertrophy	Bedtime, wake‑up, deep/light sleep, total sleep	Changes in sleep parameters before and after Twin-Block insertion (dental procedure to advance mandible and improve malocclusion/ can also alleviate sleep apnoea but not sued for that reason here) 10 days before and 10 days after the insertion.	Wearable sleep tracker (Xiaomi Mi Band 6)	Twin‑Block insertion causes immediate sleep disturbances: Bedtime shifted from 21:30 (20:41–21:48) to 00:15 (23:44–00:26) (p < 0.001); Wake‑up from 6:48–9:28 (p < 0.001); Light sleep increased from 5:42–6:04 (p = 0.009); Sleep duration and deep sleep unchanged.
Rossouw *et al* [60]	2024 South Africa	Randomised control trial (Sleep Intervention) n=60 (30 intervention and 30 in control group) age = 16.1 (1.0) years girls= 75% Ancestry split: 3% European ancestry 62% Mixed ancestry 35% African ancestry (no group difference in demographics) Exclusion criteria: substance use, traumatic brain injury, pharmacotherapy/ psychotherapy for PTSD	Sleep quality	Post-traumatic stress disorder	Questionnaire: PSQI	The South African Adolescent Sleep Intervention (SAASI) intervention was effective in reducing PTSD symptoms but did not show a clear advantage in improving sleep quality
Yaria *et al* [61]	2024 Nigeria	Case report n: 1 Age: 17y Sex: M	Narcolepsy	Mean sleep latency, CSF hypocretin level, excessive daytime sleepiness, cataplexy, sleep paralysis	EEG CSF hypocretin level, clinical symptoms	Narcolepsy diagnosis and management remain challenging in Nigeria due to limited resources, lack of awareness, and unavailability of medications.
Bougrine *et al* [62]	2024 Tunisia	Longitudinal (repeated measures over 6 phases) n: 23 age: 17.2 ± 0.5y; sex: 100% F.	Sleep quality and duration	Sleep quality, Intermittent fasting, cognitive function. psychomotor performance, dietary intake during fasting.	Questionnaire: PSQI	Ramadan fasting results in cumulative fatigue and sleep loss which impairs cognitive and psychomotor performance.
Bougrine *et al* [63]	2024 Tunisia	Randomized counterbalanced crossover Comparing Early (meal between 22:30 and 23:30) and Late Suhoor (meal needs to be taken between 02:30 AM-03:30 AM) n: 26 age: 15.9 ± 0.9 y sex: 100% F	Sleep quality and duration	Sleep quality, cognitive function. Meal timing during fasting. Suhoor refers to the timing of the pre-dawn meal before the start of the daily fast during Ramadan. Comparison between Early and Late Suhoor. Outcome variables: Cognitive tests: reaction time, digit cancellation test (attention test), mental rotation test. Daytime dysfunction: one the seven components of the PSQI	Questionnaire: PSQI	Average sleep time was 7.8 hours Pre-Ramadan, but dropped to 6 hours during Ramadan. Significant increase in daytime dysfunction and fatigue with afternoon cognitive impairment was highly prevalent, especially in Early Suhoor
Youssry *et al* [64]	2024 Egypt	Cross-sectional n: 60 children with SCD and 60 controls age: 8–18 years (mean 11.4 ± 2.71 y) sex: 58.3% M.	Sleep Disordered Breathing (SDB)	Obstructive sleep apnea in children with sickle cell disease.	Questionnaires: Pediatric Sleep Questionnaire (PSQ) and Polysomnography (PSG)	83.3% of SCD children had symptoms of SDB. 73.3% were diagnosed with obstructive sleep apnea
Innocent *et al* [65]	2024 Uganda	Cross-sectional N: 548 Age: 10.3 ± 2.6 [6–17]	Sleep disturbances	Electronic use before bed, lack of parental interaction, and caregiver unemployment	**Questionnaire:** Sleep Disturbances Scale for children (SDSC) 26 questions which regroup in 6 major disorders of sleep: 1.Disorders of initiating/maintaining sleep 2. Sleep breathing disorders 3. Disorders of arousal 4. Sleep-wake transition disorders 5. Disorders of excessive somnolence 6. Sleep hyperhidrosis SDSC total score made up of those 6 disorders of sleep is then split into presence vs absence of sleep disturbances	High Prevalence of Sleep Disturbances: 21.7%. Sleep breathing disorders prevalence 95%CI [18.5%−25.4%] Association of presence of sleep disturbances in multivariable logistic regression with: 1) sleep breathing disorders, 2) technology use and 3) short sleep duration. Use of electronic devices before bed doubled the risk of sleep disturbances. Children who reported sleeping <7 hours were nearly three times more likely to experience sleep problems. Less parental interaction before bedtime increased sleep disturbances.
Kerkeni *et al* [66]	2025 Tunisia	RCT N:36 Age: 8–12 y 3 groups (12 per group): Controls Soccer alone Soccer + kiwifruit (2/day, 1h before bedtime, with early dinner)	Sleep quality, Total sleep time, Sleep efficiency	Effect of kiwifruit (high levels of serotonin) consumption and soccer training on sleep quality	Questionnaire: PSQI Actigraphy	Combining kiwifruit consumption with soccer training significantly improved sleep quality, sleep efficiency, latency, and total sleep time compared to soccer alone or control.
Olorunmoteni *et al* [67]	2025 Nigeria	Cross-sectional n: 170 age: 13–19 y sex: 54.6% M	Bedtime, Wake time, Time-in-bed, Total sleep time, Sleep onset latency, Sleep efficiency, WASO, Number of awakenings	Weekend-weekday differences Rural-urban characteristics	Actigraphy	Urban and rural adolescents had insufficient sleep during the weekdays (<7h) and a catch-up sleep on weekends. This was more common among urban adolescents.

n: sample size; OSA: Obstructive Sleep Apnea Syndrome; CSHQ: Children Sleep Habits Questionnaire; ESS: Epworth Sleepiness Scale; PSQI: Pittsburgh Sleep Quality Index; ISI: Insomnia Severity Index; SDSC: Sleep Disturbance Scale for Children; SCA: Sickle cell anaemia; ADHD: Attention Deficit Hyperactivity Disorder; BMI: Body mass index; LD: Learning Difficulty; y: years; h: hours; M:Males; F: Females.

**Fig 2:**
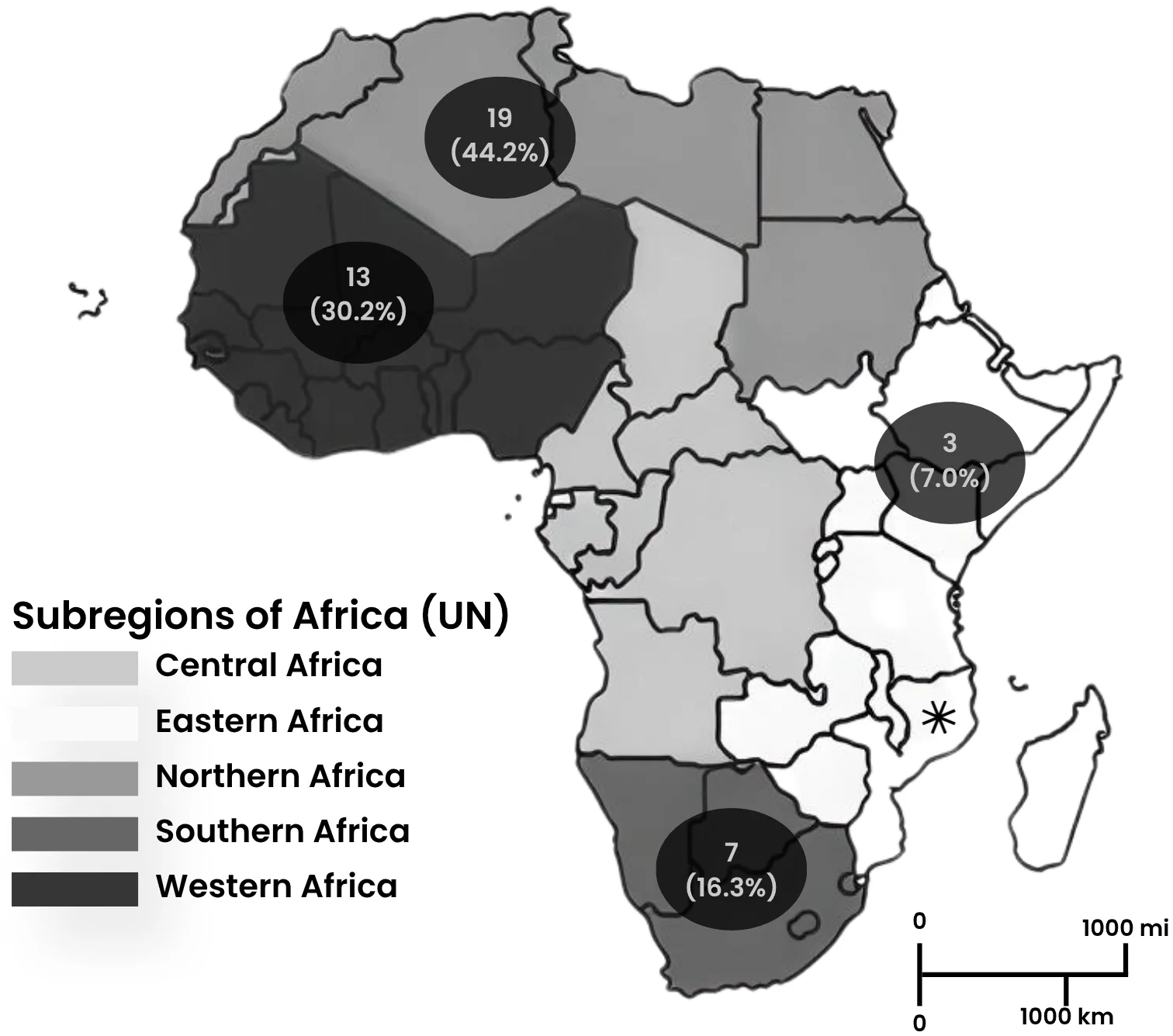
Distribution of studies on adolescent sleep across the African regions. The map illustrates the five primary African macro-regions according to the United Nations geoscheme. (*One study jointly conducted in Cape Verde and Mozambique).

**Fig 3:**
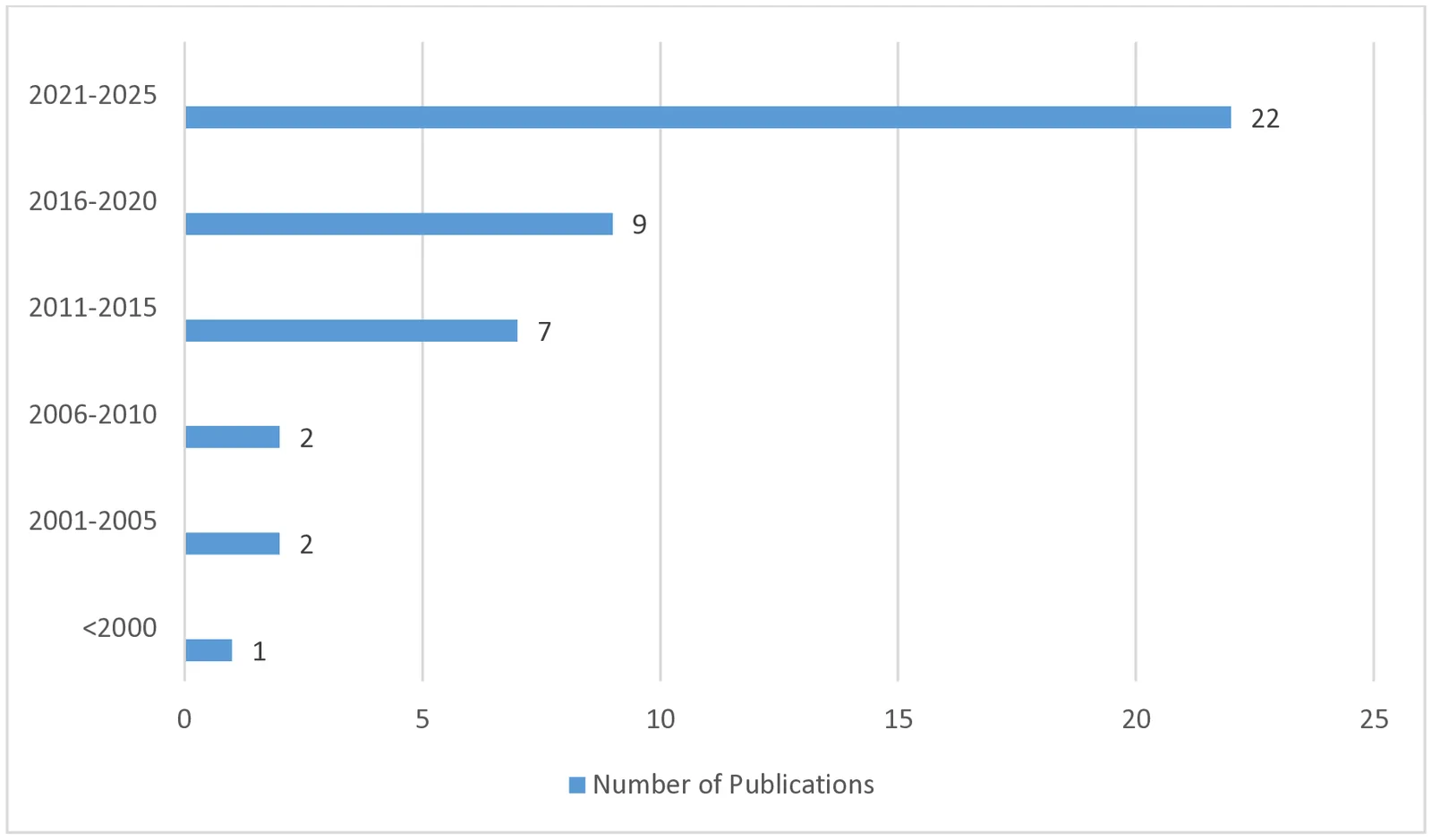
The time trend of the number of publications on Adolescent sleep in Africa.

### Types of sleep health studied and prevalence of sleep problems

The most studied sleep health dimensions among adolescents were sleep duration (13; 41.9%) and sleep quality (10; 32.3%). Nine studies (29.0%) examined daytime sleepiness among African adolescents, while four studies (12.9%) focused on sleep-disordered breathing and two studies on sleep regularity or circadian rhythms. Some of the studies reported findings on multiple sleep health measures, as shown in Table 3. The mean sleep duration reported in most studies was less than the recommended 8–10 hours for adolescents [68]. Also, there was weekday-to-weekend variability in the adolescents’ sleep duration as reported by some of these African studies (Table 3). Maduabuchi *et al*, in Southeastern Nigeria, reported a significant increase (p < 0.001) in sleep duration on weekends to a mean of 8.7 (2.1) hours from a weekday mean of 7.8 (1.9) hours [13]. Using the adolescent sleep habits questionnaire, Olorunmoteni *et al* in Southwestern Nigeria reported a mean weekday sleep duration of 7.25 ± 0.86 hours, with short sleep duration on weekdays among 44.4% of the respondents, while only 5.5% of the adolescents had insufficient sleep on weekends [15]. Among adolescents in Northern Nigeria, Peter *et al* reported a similar sleep duration ranging between 7.13 and 7.19 hours, though the weekday-weekend difference in sleep duration was not investigated [41]. Sanya *et al* in northcentral Nigeria reported a mean sleep duration of 9.3 ± 2.3 h during school days with no report of the adolescents’ weekend sleep duration. In South Africa, Reid *et al* studied the sleep behaviour of adolescents on a school day through specific questions on their sleep characteristics in the preceding night [31]. Sleep duration was assessed by determining the time in bed (TIB) [31]. The adolescents reported significantly shorter (p < 0.001) TIB during weekday school nights (453 ± 70 minutes, i.e., 7.5 ± 1.2 h) when compared to weekend nights (476 ± 128 minutes, i.e., 7.9 ± 2.1 h) [31]. The sleep insufficiency experienced by adolescents on school nights was further shown by their reported mean sleep onset latency of 17 minutes, with 45% sleeping within ten minutes, probably because of a high sleep pressure [31]. The mean total sleep time for a study involving adolescents in Morocco was 8.0 ± 1.5 hours [50]. In another Moroccan study, Aalouane *et al* reported a mean sleep duration of 7.8 hours on weekdays, 9.6 hours on weekend nights and 9.5 hours during the summer holidays [29]. There was no other study on the sleep pattern of adolescents during holidays. In total, the available studies on sleep duration showed insufficient sleep during school nights with a tendency to catch up sleep during weekends or holidays.

**Table 3:** Risk and protective factors for sleep health and the association of sleep health with academic performance, mental health and cardiometabolic risk among adolescents in Africa.

References	Year Country Study design Sample size Age Sex distribution	Risk and protective factors • Sociodemographic factors • Electronic device use • Adverse childhood experiences	Association of sleep with - Academic Performance - Mental Health and - Cardiometabolic Risk	Association of sleep disorders with - Interventions and - Medical conditions
Bénéfice *et al* [32]	2004 Senegal Longitudinal (three consecutive years), Cohort n: 40 age: 13.3 ± 0.5 at baseline sex: 100% F	**Puberty:** No association between sleep variables and puberty status **Season:** Earlier bedtimes, waketimes and shorter sleep duration were found in June (dry and earlier sunset season) than in March suggesting an association between sleep and seasonal change of daylight. No association between sleep habits and pubertal status	Significant relationship between BMI and sleep habits. Girls with lower BMI had longer sleep.	---
Aalouane *et al* [29]	2011 Morocco Cross-sectional n: 1451 sex: 56.7% M	**Sex:** Females had earlier waketimes, more WASO and more nightmares.	About half of the adolescents thought that the quality of their sleep influenced their academic performance	---
Fakier&Wild [35]	2011 South Africa Cross-sectional of adolescents with and without learning difficulties (LD) n: 703 age: LD:13–20 Control: 14–18 sex: LD: 66% M Control: 47% M	**Substance use**: Sleep problems independently associated with tobacco, cannabis and inhalant use when learning difficulties were taken into account.	Adolescents with learning difficulties had more sleep problems	---
Alabi *et al* [36]	2012 Nigeria Cross-sectional n: 909 age: 3–16 sex: 50.0%F	---	Daytime sleepiness during class and poor memory more prevalent in snorers Obesity: present in 33.5% of snorers	---
Borchers & Randler [37]	2012 Côte d’Ivoire Cross-sectional n: 588 age:12.7 ± 1.6 [10–15] sex: 57.5% F	Sex was the most influential factor for sleep variables with 9.9% of variance explained on Partial eta-squares followed by residence (8.3%), age (6.2%), religion (4.5%) occupation (2.9%). **Sex**: Girls got up earlier and had an earlier midpoint of sleep (weekdays & weekends) **Residence:** Urban adolescents had later waketimes, earlier bedtimes, longer sleep duration and later midpoint of sleep on weekdays. **Age:** Earlier wake times, later bedtimes and later midpoint of sleep on weekdays among older adolescents. On weekends, older adolescents had later bedtimes but wake times remained similar **Religion:** Muslims had earlier waketimes on weekdays and weekends compared to non-Muslims. **Occupation:** In-school adolescents had later waketimes and longer sleep duration on weekends than out-of-school (working) adolescents.	---	---
Maduabuchi *et al* [13]	2014 Nigeria Cross-sectional; n: 443 age: 13–19 sex: 59.4% F	**Sex** did not significantly influence the sleep onset latency (χ2 = 32.89, p = 0.57). **Weekday-Weekend difference:** There was a significant difference in the mean sleep duration (t = 6.12, p < 0.001) probably due to long school hours on weekdays, extra classes **Religion:** Religious activities suggested as affecting adolescent sleep habits but no quantitative data on this.	---	---
Sanya *et al* [39]	2015 Nigeria Cross-sectional n: 1033; age: 12–19 (15.3 ± 1.6); sex: 51.7% F	**Age**: Older teenagers had later bedtimes and waketimes **Sex** did not significantly influence sleep habits.	---	---
Balogun *et al* [12]	2017 Nigeria Cross-sectional n: 450; age: 10–18(13.4 ± 1.2); sex: 52% F	**Sex and age:** No significant association with self-rated sleep quality. Higher proportion of respondents with good sleep quality (70.8%) in junior secondary school.	---	---
Christoph *et al* [40]	2017 Uganda Cross-sectional n: 148 age: 11–16 (12.1 ± 1.1) sex: 61.8% M	**Residence:** (rural/urban) not associated with a difference in self-reported sleep duration and subjective sleep quality	Lower BMI percentile was associated with being female, living in a rural community, being physically active and having better subjective sleep quality	---
Peter *et al* [41]	2017 Nigeria Cross-sectional n: 353 age: 15.89 ± 1.93 sex: 61.8% M	**Screen use:** 62.9% watched TV or played video games, and 42.8% used social media, both associated with delayed sleep onset and reduced sleep quality. 73.4% were exposed to **generator noise** till bedtime 31.2% consumed stimulants 27.8% felt hungry at bedtime	---	---
Regaieg *et al* [42]	2017 Tunisia Cross-sectional n: 1695 age: 15–18 years sex: 56.3% F	**Age:** 72.5% of older (**15–18 years)** adolescents had short sleep duration (<8 hours/day) **Sedentary lifestyle:** 75.4% of those with sedentary activities of >4 hours/day had short sleep duration	The prevalence of obesity and the waist circumference value was significantly higher for short sleepers as compared with normal sleepers (p = 0.04 and p = 0.05) respectively)	---
Cheikh *et al* [43]	2018 Tunisia Randomised (double-blind crossover design) clinical trial n: 10 Teenage athletes; age: 15.4 ± 0.3 sex: 100.0% M	---	Better subjective sleep quality, reduced fatigue, and muscle soreness after MEL ingestion compared to placebo.	Nocturnal ingestion of melatonin (MEL-10 mg) was significantly associated with higher TST and sleep efficiency (SE), and lower SOL and nocturnal WASO. MEL administration resulted in higher durations of stage N3 sleep and REM sleep while reducing N1- and N2-sleep stage durations.
Olorunmoteni *et al* [15]	2018 Nigeria Cross-sectional n: 346 age: 10–19 (13.5 ± 2.29) sex: 51.4% F	**Screen use:** Significant association between higher nighttime use of computers, cell phones, TVs and shorter sleep durations. Lower social class and public school attendance are associated with longer weekday sleep duration; **Female sex**, **younger age,** and living in a polygamous family are linked to longer sleep on weekends. Room sharing in 74.6%, but not associated with sleep duration on weekdays or weekends	---	---
Carneiro *et al* [44]	2019 Cape Verde (CV) & Mozambique (M) Cross-sectional n: 651 age: 2 -15y &4-13y 2 – 7Y: CV: 61%; M: 47% 8 – 10y: CV: 50%; M: 40% 11–15 CV: 15%; M: 13% sex: 51% F	**Screen use:** Both samples showed an association between watching television to fall asleep and higher CSHQ scores, indicating that electronic use before bed is associated with sleep disturbances Higher rates of co-sleeping were found among the Cape Verde sample than the Mozambique sample (63% vs 29%)	---	---
Hanson et al [45]	2019 South Africa Longitudinal n: 1414 age: 12–17 sex: 52.7% F Group-based multi-trajectory modelling was done to group the participants into classes based on longitudinal trajectories (time spent in physical activity/ sports, sedentary behaviour, and sleep on weekdays and weekends)	**Maternal education:** was significantly associated with sleep trajectories (Adolescents whose mothers had secondary school education were more likely to be in the 7h or 8h per night sleep trajectory compared to the 9h per night trajectory)	Sedentary behaviour and sleep trajectories did not cluster together with the physical activity domains (an adolescent with short sleep and high screen time might not also be someone with low physical activity).	---
El-Baroudy *et al* [46]	2020 Egypt Cross-sectional n: 80 (40 children on regular hemodialysis, 40 controls) age: 6–15 sex: Patients: 65% F Controls: 55% F	---	---	Higher prevalence of sleep disorders in children with end-stage renal disease (ESRD) compared to healthy children
Abdelmaksoud *et al* [47]	2021 Egypt Case-control n: 120 (60 Type 1 Diabetes mellitus, 60 controls) age: 10–18 (13.57 ± 1.76) sex: TID: 30% F Controls: 36% F	---	There was a negative correlation between insulin sensitivity score and total sleep time (*p* = 0.002); and a positive correlation with sleep efficiency (*p* < 0.001). There was a **negative correlation between glycated haemoglobin levels and sleep efficiency (*p* < 0.001) and REM sleep (*p* = 0.003)** and a positive correlation with sleep onset latency (p = 0.005)	T1D + microvascular complications associated with lower sleep efficiency and less REM sleep Worse sleep quality is associated with poorer glycemic control, higher insulin resistance and the presence of microvascular complications in TID
Micklesfield *et al* [48]	2021 South Africa Longitudinal association between school night sleep between ages of 12–18 and anthropometric measurements at 18 years old (adjusting for physical activity and sedentarity) n: 1337 age: 12–18; sex: 52% F	**Sex differences in sleep patterns:** - **Males:** 35% slept 8 hours or less on school nights - **Females:** 65% slept 8 hours or less on school nights	In adjusted analysis, **male adolescents who slept ≥9 h/night,** compared to those who slept ≤ 8 h/ night on weekdays had significantly **lower** **-BMI** β [95%CI]= -1.0 [-1.4 to -0.5] **-Waist circumference** β [95% CI]=-2.3 [-3.7 to -1.1] **- %Body fat** β [95%CI]= -1.5 [−2.8 to −0.1] There was no association between weekday sleep in females and anthropometry and body composition.	---
El Refay *et al* [49]	2021 Egypt Cross-sectional n: 765 age: 4–16 sex: 53.3% M	**increased screen time:** One of the factors associated with the prevalence of sleep disorders	Significant positive correlation was found between family income affection, SDSC, and anxiety (p = 0.001 and 0.004)	Psychological stress, Anxiety symptoms
Wolde *et al* [20]	2022 Ethiopia Cross-sectional n: 799 age: 10–19 sex: 52.1% M	**Substance use:** Adolescents with inhalant use disorder (AOR = 1.98), internet addiction (AOR = 3.33), and khat use disorder (AOR = 1.74) were positively associated with PSQ.	Adolescents with physical illness had about 4 times higher odds of association with PSQ (AOR = 3.76	--
Moustakbal & Maataoui [50]	2022 Morocco Cross-sectional n: 730 age: 15.64 ± 1.66 sex: 55.5% F	**Age:** The prevalence of poor sleep quality increases with increasing age (58% for early adolescents, 76% for middle adolescents and 84% for late adolescents. **Screen use:** Shorter TST in mobile phone users (7.37 ± 1.50 h) than non-users (8.22 ± 1.38 h) (*p* < 0.001). **Health**: Poor sleep quality is more in adolescents with physical or psychological health issues **Residence:** Adolescents in urban areas had: **•** Better sleep quality 66% vs 77% (*p* = 0.002) **•** Later bedtime (11:15 pm vs 10: 30 pm: (p < 0.001) **•** Later wake time (7:51 am vs 7:08 am (*p* < 0.001) **•** No significant difference in TST (p = 0.623)	There was no significant difference between sleep duration and categories of BMI (underweight, normal and overweight) though adolescents with overweight had a lower sleep duration (7.35 ± 1.43 h).	---
Elfaki *et al* [51]	2023 Sudan Cross-sectional n: 921 age: 10–15 sex: 57.9% F	**Age:** Higher prevalence of short sleep duration (<8h) in early adolescents: 62.09% **Gender:** No significant difference in short sleep duration between boys and girls **Residence:** No significant difference in short sleep duration between rural and urban dwellers	Higher prevalence of short sleep among overweight and obese adolescents (p < 0.05). The prevalence of metabolic syndrome (MetS) among short sleepers was 2.8% with the highest risk of MetS observed at less than 6.5 hours of sleep per night	---
Guembri *et al* [52]	2023 Tunisia Cross-sectional n: 72 age: U14:13.4 ± 0.5y U17: 16.3 ± 0.5y. sex: 100.0% F	**Age:** No significant differences were shown between U14 and U17 players in sleep quality, insomnia and daytime sleepiness scores	---	---
Mlouki *et al* [53]	2023 Tunisia Cross-sectional n: 404 age: 16–18 (17.18 ± 1.5) sex: 63.0% F	Significant link between ACEs and sleep disorders, **Internet addiction:** Partial mediator in the relationship between ACEs and sleep disorders Cyber addiction had a major mediation effect among females (% mediation = 30%)	Elevated risk of sleep disturbances in adolescents facing more adversities	---
Olorunmoteni *et al* [54]	2023 Nigeria Cross-sectional n: 448 age: 10–19 sex: 57.8% F	**School schedule and school type**: Statistically significant association between difficulty in concentrating in class and sleep quality as well as sleep duration on both weekdays (*p* = .012) and weekends (*p* = .031) Earlier school closing times were associated with better sleep quality. Attending a private school, longer school hours or extra activities was associated with poor sleep quality	Depression showed statistically significant association with poor sleep quality (p < .001)	---
Tantawy *et al* [55]	2023 Egypt Cross-sectional n: 20 age: 6–18 (10.22 ± 3.28) sex: 63.3% M	---	---	Sickle cell anaemic children with moderate to severe OSA had significantly higher tricuspid regurgitation, velocity (p = 0.007) and left middle cerebral artery flow velocity (higher stroke risk) (p = 0.049) when compared to those with AHI < 5
El‑Jaziz *et al* [56]	2023 Morocco Prospective cohort with repeated measurements N: 50 Age: 17.22 ± 1.15 y Sex: 66% F	**Risk** Age: mean 17.22 ± 1.15 y (adolescent developmental stage) Evening chronotype: Fasting schedule disruption made VO_2_ peaked downwards from 46.82 to 39.15 ml/kg/min **Protective** Morning chronotype: n = 47 (94%) Regular training despite fasting Shared suhur/iftar and prayer times	---	---
Olorunmoteni *et al* [57]	2024 Nigeria Cross-sectional N:900 Age: 13–19 59% female	**Risk Factors** 79% overall insufficient sleep; urban adolescents average 6.55 h/night vs. rural 7.23 h/night (p < 0.001) Urban youth go to bed significantly later than rural peers (p < 0.001) Single‐parent households linked to poorer sleep quality and higher apnea risk Later school end times associated with poor sleep quality & daytime sleepiness Electronic device use (higher in urban areas)	---	**Medical conditions**: Risk of sleep apnea: overall 9.8%; urban 12.6% vs. rural 7.1% (p = 0.006)
El Mlili *et al* [58]	2024 Morocco Cross-sectional n:60 age: 6–17 y girls: 75%	**Risk Factors** Age: mean 11.05 ± 0.35 y Gender: 44.4% of girls vs. 33.3% of boys had sleep disability Chronic illness: T1DM as physiological stressor **Protective Factors** Lower HbA1c associated with fewer psychological symptoms (p = 0.0001) All participants under insulin therapy (structured routine)	26.6% of girls vs. 20% of boys had anxiety; 11.1% of girls vs. 40% of boys had ADHD symptoms Salivary cortisol (stress biomarker) correlated with anxiety (p = 0.02) and poorer glycaemic control (HbA1c, p = 0.0001)	**Medical conditions**: T1DM in all participants; higher cortisol levels in girls (mean 5.2 vs. 4.2 ng/mL; p = 0.004)
Saifeldin *et al* [59]	2024 Egypt Intervention study: Twin Block insertion for mandibular malocclusion. n: 24 age:11–13 y sex: 100%	**Risk Factors** Young females (11–13 y) Bedtime delays by ~2 h 45 min; wake-up delayed by ~2 h 40 min (p < 0.001) **Protective Factors** High compliance with orthodontic intervention worn at ≥17 h/day No further sleep decline after 5 days	No data reported	Twin-Block appliance: Immediate impact on sleep patterns – Bedtime: median 21:30–00:15 (IQR 23:44–00:26) – Early wake-up times – Light sleep ↑ from 5 h 42 m to 6 h 04 m (p = 0.009) – Deep sleep & total sleep: no change (p > 0.48) – 83.3% reported noticeable sleep changes
Rossouw *et al* [60]	2024 South Africa Randomised control trial (Sleep Intervention) n=60 (30 intervention and 30 in control group) age = 16.1 (1.0) years girls= 75%	Risk Factors **•** Trauma exposure & PTSD: All participants had PTSD (CPSS-5 ≥ 21) and sleep disturbances at baseline • Insufficient sleep: Only 39.3% slept >7 h/night pre-intervention • Fear of sleep: Elevated FoSI scores indicating high sleep-anxiety • Adolescent stage: 15–19 y, a period of heightened vulnerability to stress Protective Factors • SAASI group format: Group-based sleep hygiene and cognitive restructuring • Non-specialist delivery: Intervention delivered effectively by nurses/counselors • Structured sessions: One individual + four group sessions providing social support and skills practice	Mental Health: – PTSD: Scores reduced from 43.9 to 27.0 (post) & 30.5 (1 mo FU) in intervention vs. 46.5 → 41.9 → 31.5 in control (p < 0.05). – Depression: KADS-6 scores improved significantly in intervention (p = 0.007). – Fear of sleep: FoSI decreased in intervention (p = 0.03).	Intervention Effects • PTSD: Post-treatment PTSD prevalence 16.7% vs. 55% in control (p = 0.011). • Sleep Quality: PSQI scores declined in both groups but no significant between-group difference (p = 0.583). • Adherence & Feasibility: Dropout 32% (intervention) vs. 26.7% (control); high initial engagement but retention challenges. Medical conditions • PTSD and co-occurring sleep disturbance were the clinical focus; no other medical comorbidities assessed.
Yaria *et al* [61]	2024 Nigeria Narcolepsy case study (n = 1)	Risk Factors • Adolescent male (17 y) in a resource-limited tertiary center with minimal sleep-medicine awareness. • Diagnostic delay: 2 yr from symptom onset vs. global median 8–10 yr. • Misdiagnosis: initially treated as epilepsy—reflects low provider recognition (global misdiagnosis ~60%). • Access barriers: no local polysomnography; CSF hypocretin test sent overseas, delaying care. Protective Factors • Behavioral modifications: scheduled naps and sleep hygiene instituted in absence of stimulants.	---	Interventions • Behavioral: scheduled naps, sleep hygiene routines. • Pharmacologic: fluoxetine for cataplexy; Modafinil unavailable. Medical Conditions • Narcolepsy Type 1: EDS despite 7–8 h sleep; cataplexy triggered by laughter; sleep paralysis. • Objective findings: MSLT latency 6 min 39 s; CSF hypocretin < 30 pg/mL.
Bougrine *et al* [62]	2024 Tunisia Longitudinal (repeated measures over 6 phases) n: 23 age: 17.2 ± 0.5y; sex: 100% F	Risk Factors • Age & Sex: 26 adolescent female athletes, mean age 15.9 ± 0.9 y (heightened fasting vulnerability) • Sleep loss: TST ↓ from 7.8 h Pre-Ramadan to 6.0 h during Ramadan (−23%) • Daytime dysfunction: significant increase in reported fatigue and sleepiness Protective Factors • Late Suhoor timing: mitigated afternoon cognitive decline • Athletic training: may have supported resilience in morning/midday cognitive tasks • Structured routine: consistent training environment anchoring sleep–wake cycles	Mental Health: increased daytime dysfunction and fatigue suggest impaired mood and vigilance; no formal mood scales used • Cardiometabolic Risk: body mass ↓ by ~1.5% and BMI decreased slightly (p < 0.05), indicating metabolic adaptation to fasting	Intervention Effects • Suhoor timing: Late-Suhoor vs. Early-Suhoor crossover: – Afternoon SRT, CRT, ATT, MRT scores declined significantly under Early Suhoor (p < 0.05) – Late Suhoor attenuated these declines (ηp² up to 0.14) • Medical conditions: none—healthy athletes only
Bougrine *et al* [63]	2024 Tunisia Randomized counterbalanced crossover Comparing Early (meal between 22:30 and 23:30) and Late Suhoor (meal needs to be taken between 02:30 AM-03:30 AM) n: 26 age: 15.9 ± 0.9 y sex: 100% F	Risk Factors Adolescent females (n = 26, mean age 15.9 ± 0.9 y): developmental delayed sleep phase plus fasting stress. TST ↓ from 7.8 h pre-Ramadan to 6.0 h during Ramadan (−23%). PSQI global score rose from 2.2 ± 0.9 pre-Ramadan to 7.3 ± 1.4 in week 4 (PSQI > 5 in 100% by end). Protective Factors Late‐Suhoor timing (PSQI 5.1 ± 2.0 vs. early Suhoor PSQI 7.3 ± 1.4). Consistent training routine: morning/midday performance preserved. Stable caloric intake: ~ 1,490 kcal/day maintained, buffering energy deficit.	Increased daytime dysfunction and fatigue imply mood/vigilance impairment; no formal psychiatric scales used. Small drop in body-mas (60.2 kg → 59.8 kg, −0.7%) and BMI decrease (p < 0.05) indicate energy balance shift without adverse markers.	Suhoor Timing: Afternoon SRT, CRT, ATT and MRT performance declined significantly in Early Suhoor (p < 0.05, ηp² up to 0.14) but was significantly better under Late Suhoor (p < 0.05). • No sleep disorder diagnoses assessed.
Youssry *et al* [64]	2024 Egypt Cross-sectional n: 60 children with sickle cell disease and 60 controls age: 8–18 years (mean 11.4 ± 2.71 y) sex: 58.3% M.	– Male gender associated with higher risk of OSA (p = 0.01); Older age (≥10 years) linked to moderate-to-severe OSA (p = 0.02)	Implicit cardiometabolic risk through endothelial dysfunction and elevated TNF‑α, IL‑6, IL‑17A linked to severe OSA.	Routine polysomnography screening for early detection, but no intervention trialed Strong association between obstructive sleep apnea (OSA) and sickle cell disease (SCD); OSA severity correlated with endothelial dysfunction and systemic inflammation
Innocent *et al* [65]	2025 Uganda Cross-sectional N: 548 Age: 10.3 ± 2.6 [6–17]	Children of unemployed parents were 3.31 × more likely to have sleep disturbances (p = 0.003) – Less parental interaction increased risk 1.83× (p = 0.037) – Sleeping in a hot room significantly associated with disturbances (p = 0.016) – Screen use before bed doubled the likelihood of sleep problems (p = 0.031)	---	--- Sleep Breathing Disorders (SBD) were the most common subtype (7.5%); no comorbidity with chronic illness was assessed
Kerkeni *et al* [66]	2025 Tunisia RCT N:36 Age: 8–12 y 3 groups (12 per group): Controls Soccer alone Soccer + kiwifruit (2/day, 1h before bedtime, with early dinner)	Children were overweight/obese (BMI ≥ 85th percentile), aged 8–12 years, which may indicate a metabolic risk profile tied to poor sleep.	Overweight/obese status links baseline sleep disturbance to elevated cardiometabolic risk. Improvements in sleep and physical activity likely reduce this risk.	- Small-sided soccer games (SSSG) improved sleep latency by ~11.9 min (from 82.2 to 59.5 min). - Kiwifruit + SSSG (SSSG-K) further reduced latency to 31.0 min, with sleep efficiency rising to 94.2%, and total sleep time improving from 6.2 to 8.5 hours/night (from ~371 min to ~512 min). - Exercise performance also improved (distance increased by ~800 m). Children with pre-existing sleep problems tied to overweight/obesity (no comorbidities like sleep apnea measured).
Olorunmoteni *et al* [67]	2025 Nigeria Cross-sectional n: 170 age: 13–19 y sex: 54.6% M	Adolescents aged 13–19; 170 from rural and urban settings in Osun State. Average age: 15.8 (rural) vs 15.3 (urban) (p = 0.04). Urban adolescents had significantly shorter sleep duration (mean TST = 5.85 hrs vs. 6.26 hrs in rural), also when adjusting for age and sex in multivariable analyses. Urban adolescents’ later bedtimes may be caused by electronic light exposure and screen use.	Adolescents reportedly experienced weekday sleep debt, a known risk for obesity, insulin resistance, and CMR over time.	---

n: sample size; D: Sleep disorders; DS: Daytime sleepiness; SA: Sleep architecture with polysomnography; SDB: Sleep disordered breathing; SD: sleep duration; ST: sleep timing; SV: sleep variability; SQ: sleep quality; SR: Sleep regularity; SP: Sleep pattern; OSA: Obstructive Sleep Apnea Syndrome; CSHQ: Children Sleep Habits Questionnaire; ESS: Epworth Sleepiness Scale; PSQ: Poor sleep quality; PSQI: Pittsburgh Sleep Quality Index; ISI: Insomnia Severity Index; SDSC: Sleep Disturbance Scale for Children; SCA: Sickle cell anaemia; ADHD: Attention Deficit Hyperactivity Disorder; ACEs: Adverse Childhood Experiences; BMI: Body mass index; LD: Learning Difficulty; y: years.

Sleep quality was assessed with the PSQI in seven studies (22.6%) [12,13,20,50,52–54], while four studies utilised self-reported subjective sleep quality measures including one study which used a visual analogue scale [31]. The prevalence of poor sleep quality, as defined by PSQI>5, reported among two adolescent populations studied in Nigeria was 50.2% [12] and 85% [54]. Similar high prevalence of poor sleep quality was reported in Morocco, with 72% of adolescent participants having poor sleep quality [50]. In Ethiopia, the prevalence of poor sleep quality among the 799 adolescents studied was 38% [20] while Mlouki *et al* reported a prevalence of 94% in a study involving Tunisian adolescents [53]. However, there were some other studies where PSQI was utilised, but they did not provide an overall prevalence of poor sleep quality. Instead, they described sleep problems measured by different components of the PSQI.

Among 825 South African adolescents, Reid *et al* reported that later bedtimes were associated with male gender, napping and taking medication and while later wake times were associated with higher total sleep time (TST), higher sleep onset latency (SOL), more awakenings, and later bedtimes.[31] The timing of the sleep-wake cycle/chronotype of the African adolescents was only reported by Borchers *et al* who utilised the midpoint of sleep to determine the sleep-wake cycle of 588 adolescents in Côte d’Ivoire. The midpoint of sleep on weekdays (1:26 ± 00:30) was significantly earlier than that of the weekends at 1:37 ± 00:42 (t = −9.09; *p* < 0.001) [37]. The adolescents also had an average sleep duration of over 8 hours on both weekdays (8:51 hours) and week-ends (8:42 hours) which is within the recommended guidelines for adolescent sleep duration [37]. Among a sample of 72 female handball players in Tunisia, the prevalence of poor sleep quality was reported at 61.1% for the U14 group and 63.8% for the U17 group [52]. Furthermore, excessive daytime sleepiness was reported by 19.4% of the U14 group and 22.3% of the U17 group, and mild insomnia was observed in 8.3% of U14 players and 8.4% of U17 players [52]. Thus, the majority of reports on sleep quality showed a high percentage of poor sleep quality among African adolescents.

### Sociodemographic disparities in sleep health

#### Rural-urban residence.

There were some disparities in the reported sleep patterns and sleep health components of African adolescents, especially among various socio-demographic groups and urban-rural locations. Christoph *et al,* in a sample of 148 adolescents, reported that rural Senegalese adolescents had a longer sleep duration but lower sleep efficiency compared to their urban counterparts, highlighting possible environmental influences on sleep health [40]. Also, Galal *et al,* in a sample of 863 Egyptian adolescents reported that 33.3% of urban adolescents and 29.5% of rural adolescents reported having a sleep disturbance, though this was not statistically significant [30]. In another study among Moroccan adolescents by Moustakbal [50], urban adolescents had significantly later bedtimes (11:15 pm vs 10:30 pm), sleep latency, and wake-up time (7:51 am vs 7:08 am) than their rural counterparts, but there was no significant difference in total sleep time**.** Furthermore, the study reported that adolescents from rural areas, older adolescents, and those with physical or psychological health issues were more likely to report poor sleep quality [50]. Conversely, Borcher *et al* [37] reported that in Côte d’Ivoire, urban adolescents had shorter sleep duration, evidenced by later bedtimes and earlier wake times than rural adolescents. Therefore, most of the studies reported longer sleep duration but poorer sleep quality among rural adolescents compared to urban adolescents though the pattern of bedtimes and wake times between rural and urban adolescents appear inconsistent across studies probably from differences in living conditions or contexts.

#### Sex.

Differences in sleep health and sleep behaviour by sex were reported in some studies as shown in Table 3 [14,21,26,28,29]. Egyptian adolescents showed variations in self-reported sleep perceptions, with boys perceiving their sleep health as healthier compared to girls [30]. Similarly, Aalouane *et al*, in a study of a group of adolescents in Morocco reported that females had earlier wake times, more wake after sleep onset (WASO) and more nightmares than the males, though this was not associated with lower sleep duration [29]. Also, Micklesfield *et al* reported a short school-night sleep duration of ≤8h/night among 65% of female vs. 35% of male adolescents in the South African adolescent population studied on weekdays [48]. Conversely, in Nigeria, being female was reported as an independent predictor of sufficient sleep on weekends, though both boys and girls had short sleep on weekdays [15]. Borchers *et al* [37] in a study that was conducted in Côte d’Ivoire, reported that female adolescents had earlier bedtimes, wake times, and an earlier midpoint of sleep than male adolescents. Sanya *et al* reported no sex difference in the sleep parameters (collected via questionnaires) studied among Nigerian adolescents [39]. However, in a longitudinal study of South African adolescents of the Birth-to-Twenty Plus Cohort, school night sleep decreased across adolescence in males but remained stable in female adolescents while weekend sleep duration remained stable from age 12–17 years for both genders [45]. The study found four school-night sleep trajectories in males and 3 trajectories in females, with two of these trajectories being in the insufficient sleep duration range (7 hours and 8 hours trajectories). On weekends, there were 3 sleep trajectories identified for male adolescents and 4 trajectories for female adolescents with only one trajectory below 8.5 hours of sleep duration for both genders.

#### Age.

Some consistencies emerge in the association between sleep health and adolescents’ age (Table 3). Younger age was reported to be associated with longer sleep on weekends among a population of Nigerian adolescents [15]. Shorter sleep in older adolescents was also found in Borchers *et al* in Cote d’Ivoire. They reported later bedtimes, earlier wake times, shorter sleep duration and later midpoint of sleep on weekdays with increasing age among adolescents in Côte d’Ivoire [37]. Also, they reported later bedtime on weekends among older adolescents while wake times on weekends remained similar across all age groups [37]. Similarly, Sanya *et al* reported later bedtimes and delayed wake time among older adolescents compared to the younger ones though there was no significant difference in sleep duration on weekends across the age groups [39]. Among 730 Moroccan adolescents, poorer sleep quality (using the global PSQI score) was observed with increasing age [50]. Also, 72.5% of older adolescents in a Tunisian study had short sleep duration (<8 hours/day) [42]. Overall, the younger adolescents reported having better sleep health, longer sleep duration, earlier bedtimes, earlier wake times, better sleep quality, and earlier chronotypes than the older adolescents, especially on weekends.

Other sociodemographic variables associated with sleep health in different studies include social class, parental education, adolescent’s student status (full-time work vs full-time student) and religion. Olorunmoteni *et al* (2018) reported the association of lower social class and public school attendance with longer weekday sleep duration among Nigerian adolescents, highlighting socioeconomic disparities in sleep patterns [15]. In addition, this study showed the contribution of household dynamics where living in a polygamous family or room sharing was associated with longer sleep on weekends. This may indicate varying household factors that can affect sleep routines and serve as a potential for the development of sleep disorders. Interestingly, this Nigerian study’s finding showed a reverse association compared to what is seen in the US, where socially disadvantaged people were reported to have poorer sleep health. Conversely, another Nigerian study by Olorunmoteni *et al* (2024) found single-parent family type to be associated with a higher odds of poor sleep quality, while adolescents from single-parent and separated parents had a higher odds of risk of apnoea. However, there was no significant association of the sleep health variables with polygamous family type. Carneiro *et al* found that more educated parents had children with better sleep habits in Cape Verde and Mozambique [44]. In a longitudinal study involving South African adolescents, maternal education was significantly associated with sleep characteristics [45]. Adolescents whose mother had completed secondary school education had more likelihood of being in the 7 (>3 times) or 8-hour-per-night (>2 times) trajectory compared to being in the 9-hour-per-night trajectory in the boys [45]. This suggests that higher levels of education in mothers were associated with lower sleep duration in their male adolescents Furthermore, in-school adolescents in Côte d’Ivoire slept longer and woke up later on weekends than working (and non-school-going) adolescents (p = 0.002) but there were no significant weekday differences in the sleep pattern or in-school versus working adolescents [37]. The Côte d’Ivoire study also reported that Muslim adolescents had earlier bedtimes, earlier wake times, and earlier chronotypes than Christian/ other religion adolescents, though both Muslims and Christians had shorter sleep duration on weekdays and weekends compared to adolescents of other religions (all p < 0.05) [37]. Thus, sociodemographic factors contribute to sleep health disparities in African adolescents though there were conflicting findings for many of the studied variables as shown in Table 3.

#### Pattern of disordered sleep.

The reported pattern of disordered sleep among African adolescents varied widely. Symptoms of sleep disorders reported included insomnia, excessive daytime sleepiness, sleep-disordered breathing, and different forms of parasomnias. Among 20 studies where the pattern of disordered sleep was provided or discussed, the values differed based on the component of sleep health studied or the type of sleep disorders’ classification system used. The findings in Table 3 show the prevalence of adolescent sleep problems and disorders across different regions in Africa. The prevalence of difficulty in initiating sleep (4.8%) and difficulty falling asleep (5.9%) were reported by Maduabuchi et al [13], who also reported that unusual sleep behaviours (parasomnias) were notably prevalent among Nigerian adolescents with sleepwalking in 6.3%, sleep talking in 15.1%, bruxism in 7.6%, and restless legs syndrome in 6.5% [13]. Among adolescents, sleep-related breathing disorders were reported in some countries. In Nigeria, the prevalence of snoring, assessed through self-report, was 34.2% [36]. Also 34.1% of those who were snoring reported associated restlessness during sleep, while 39.5% of the snorers exhibited cessation of breathing [36]. In another Nigerian study, 4.8% of the adolescents were aware of having sleep-disordered breathing [41]. Some studies did not provide the prevalence of specific types of sleep disorders but provided the overall prevalence of sleep disorders among the adolescent population studied. In Tunisia, a high prevalence of disordered sleep was reported in 94% of the 414 adolescents studied, though the specific types of sleep disorders were not described [53]. Also, a significant proportion (65.6%) of the 765 Egyptian adolescents studied by El Refay *et al* had symptoms of sleep disorders, with problems initiating and maintaining sleep identified as the most prevalent symptoms [49]. Thus, the most common types of sleep disorders reported among the African adolescents in this review are insomnia (disorders of initiating or maintaining sleep), parasomnias and sleep-related breathing disorders.

### Sleep disorders in adolescents with specific co-morbidities

The prevalence of sleep disorders among adolescents with specific medical conditions varied. A high prevalence of sleep disorders was reported among adolescents with end-stage renal disease (ESRD) undergoing hemodialysis as studied by El-Baroudy *et al* [46]. Among the 30 children with sickle cell disease (SCD) studied in Egypt, 60% had sleep apnea (defined as an apnea-hypopnea index of > 1 event/hour) [55]. Among the 30 children with SCD studied, Tantawy *et al* found an association between sleep apnea in the studied cohort and an increased risk of pulmonary hypertension and stroke as evidenced by abnormal cerebral blood flow velocities [55]. The children with various comorbidities studied had poor sleep health indices compared to the healthy controls studied.

Table 3 presents findings on the association of sleep disorders with specific comorbidities from various studies conducted in different countries and settings. In a study conducted by Alabi et al in Nigeria, obesity was identified as a comorbidity associated with sleep disorders in children. They found that obesity was present in a significant percentage of children with sleep disturbances. Obesity was present in 33.5% of snorers, and 17.5% showed signs of adenotonsillar hypertrophy, which can contribute to sleep disordered breathing. Abdelmaksoud *et al* in Egypt studied adolescents with Type 1 Diabetes (T1D) and highlighted a lower sleep efficiency in these individuals compared to healthy controls, emphasising the impact of T1D on sleep health.

### Risk and protective factors for adolescent sleep health in Africa

#### Description of sleep hygiene practices.

Several studies reported individual-level factors such as sleep hygiene practices among adolescents as shown in Table 3. These included bedtime screen use such as watching television to fall asleep, higher consumption of caffeinated beverages, and the use of sleeping pills at night, all potentially contributing to sleep disturbances [31,44]. In Nigeria, 80.9% of adolescents were reported to use electronic devices before sleep in a cohort of urban-dwelling adolescents [15]. Another study in northcentral Nigeria (Sanya *et al* in 2014) reported that 10% of teenagers make phone calls at night while 5.5% browse the internet and use computers at night, probably reflecting variations in accessto electronic devices and/or internet services. Peter *et al* in a study conducted in North western Nigeria in 2017 reported internet use for socia media in 42.8% of adolescents till <one hour before bedtime. In a comparative study between adolescents in Cape Verde and Mozambique, higher rates of co-sleeping were found among the Cape Verde sample than the Mozambique sample (63% vs 29%) with a higher prevalence of sleep problems also in the Cape Verde sample [44]. Furthermore, the consumption of stimulants (chocolate/coffee/any other stimulant) at night was reported in one-third of a cohort of Nigerian adolescents especially among late adolescents (49.1%) [41]. Similarly, Reid *et al* reported caffeinated beverage consumption on the day before the study in 72% of the 825 South African adolescents (though not significantly associated with their sleep health) studied while 8% of them regularly used sleeping medications to enhance their falling asleep at night though the amount and type of medication use was not stated [31]. Among Ethiopian adolescents, inhalant use disorder (AOR = 1.98) and khat use disorder (AOR = 1.74) was reported to be associated with poor sleep quality [20].

#### Nighttime electronic device use.

The data from various studies on the association of sleep health with nighttime electronic use revealed varying degrees of association between late-night electronic device use and adolescents’ sleep health (Table 3). Across the studies by Carneiro *et al*, Moustakbal *et al*, Peter *et al* and Olorunmoteni *et al*., nighttime use of electronic devices was common among adolescents with the prevalence of bedtime television use being 30% in Mozambique [44] and 33% in Cape Verde [44], while electronic device use within one hour of bedtime was reported as 78% among Moroccan adolescents [50], as well as 62.9% (Peter *et al,*2017) [41] and 80.9% (Olorunmoteni *et al,* 2018) [15] in two Nigerian studies. Some of the studied adolescents were reported to frequently use social media within one hour of bedtime [41]. Peter *et al* also reported that the adolescents’ use of electronic devices within an hour of bedtime was more common among late adolescents, but this did not show any significant association with sleep problems [41]. Olorunmoteni *et al,* (2018) however reported that non-use of the screen at bedtime was an independent predictor of good sleep duration among the in-school adolescents studied. Moustakbal *et al* reported a statistically significant association between the use of mobile phones late at night and poor sleep quality, measured with PSQI (*p* = 0.001) [50]. Among Ethiopian adolescents, internet addiction contributed to increased odds of poor sleep quality [20]. These studies highlight an increasing access to and use of electronic devices such as television, mobile phones and video games before sleep over the years among the adolescents though only a few studies explored an association between screen use and sleep disturbances in the adolescents.

#### Physical activity.

The association between physical activity and adolescent sleep health was considered in some studies. Garnier et al, in a study involving 80 Senegalese adolescents using accelerometry, found that girls were more inactive than boys (4 hours 23 minutes versus 2 hours 49 minutes) [33]. They found an association between shorter durations of sleep, poorer sleep quality and more physical activity in the boys, which they hypothesized may be due to physical activity in boys being done at night and being more strenuous than that in the girls.. In South Africa, Hanson *et al* studied physical activity and the pattern of sedentary lifestyle of 1414 adolescents in their longitudinal study cohort and found that sedentary behaviour and sleep trajectories were not associated with the physical activity domains [45]. In contrast, in another South African longitudinal study, Micklesfield *et al* with the birth-to-twenty-plus cohort, reported an association between higher physical activity, sufficient sleep and more favorable body composition outcomes [48].

### Adverse childhood experiences

The study by Mlouki *et al* conducted in Tunisia among in-school adolescents revealed a significant association between adverse childhood experiences (ACEs) and sleep disorders [53]. The research highlighted that adolescents who had faced adversities were at higher risk of experiencing sleep disturbances (Table 3). The study also showed ACEs-predicted sleep disturbances with a mediation effect of internet addiction (% mediation = 18.3%, p = 0.005), particularly in female adolescents (% mediation = 30%) [53].

### Household and environmental factors

Aside from co-sleeping as a factor that can contribute to adolescents’ sleep problems, sleeping in a noisy environment was also reported as a likely barrier to healthy sleep. In the study by Peter *et al*, the main source of environmental noise was the noise from a power-generating set which was reported in 73.4% of the adolescents [41]. The contribution of other environmental factors such as household overcrowding, neighbourhood crimes and access to electricity did not appear well studied.

### School schedules

Two studies from Nigeria reported on the association of sleep health with school schedules among adolescents (Table 3). Olorunmoteni *et al* (2023) found that later school closing times and private school attendance were associated with poorer sleep quality [54]. The majority of the adolescents also (81.7%) reported having difficulty concentrating in class due to poor sleep quality- associated daytime sleepiness [54]. Furthermore, 52% of adolescents in a Moroccan study reported that preoccupation with school-related demands contributed to their sleep disturbances [29].

### Association between sleep and academic/cognitive performance and mental/ clinical health

#### Sleep health and academic performance.

The data from Table 3 presents a diverse array of studies examining the relationship between sleep disorders and academic performance across different regions and populations. Despite the variations in study design, settings, and demographics, certain similarities emerge from the findings. Alabi *et al* [36] (Nigeria) found that daytime symptoms like falling asleep during class and poor memory were more prevalent in snorers, with these symptoms contributing to disrupted sleep and a resultant poor academic performance. Moreover, the study by Fakier *et al* in South Africa highlighted that adolescents with learning difficulties had more sleep problems than those without learning difficulties [35]. Also, adolescents who had more sleep problems were more likely to use substances such as tobacco, alcohol, methamphetamine, cannabis, inhalants, cocaine, and ecstasy. with a negative effect on their academic performance [35]. This suggests a vicious cycle of relationship between sleep problems, academic performance and substance use. Aalouane *et al* also reported an association between short sleep duration and poor academic performance [29]. While some studies emphasize the detrimental effects of sleep disorders on academic performance, others highlight the importance of good sleep quality for better academic outcomes. This was supported by about half of a cohort of Moroccan adolescents who reported that the quality of their sleep influenced their academic performance [29].

### Sleep health and mental health of adolescents

The study by Olorunmoteni *et al* conducted in Nigeria among in-school adolescents aged 10−19 years found a significant association between poor sleep quality and depression severity (assessed using the Patient Health Questionnaire-9) [54]. Among adolescents with prior ACEs, a threefold increased risk of sleep disturbances was observed for those experiencing three or four ACEs while those experiencing at least five ACEs had increased the adjusted odds of sleep disorders by 11.9 folds [53]. Furthermore, internet addiction was reported to mediate the association between ACE and sleep [53].

### Sleep health and cardio-metabolic risk

The data from Table 3 shows findings from various studies on the association of sleep health with cardio-metabolic risk (CMR) factors such as basal metabolic rate (BMR), physical activity, and body mass index (BMI) in different adolescent populations across Africa. The studies reveal both similarities and contradictions in their results. Alabi *et al.* from Nigeria found a significant association between snoring and obesity [36]. Furthermore, Micklesfield *et al* in South Africa, associated consistent physical activity and sufficient sleep with more favourable body composition outcomes, including lower BMI [48]. Similar findings of association of longer sleep and lower BMI was found among girls in Senegal studied over three years [32]. Furthermore, the study by ElFaki *et al* in Sudan highlighted a significant positive association between reporting short sleep duration and the presence of metabolic syndrome components among early adolescents [51]. Tantawy et al. in Egypt, found an association between higher obstructive sleep apnea severity and worse cerebrovascular health markers in sickle cell disease patients with moderate to severe OSA [55]. This finding contrasts with the study by Christoph *et al*. in Uganda, which associated higher BMI percentile with being female, living in a rural community, having a higher physical activity frequency and better subjective sleep quality, though the overall BMI in their study was quite low such that higher BMI may actually reflect better health, rather than detrimental health in certain contexts [40]. In Tunisia, Regaieg *et al* [42] reported a higher prevalence of obesity (based on BMI and waist circumference) among adolescents with short sleep duration. The data from these studies demonstrate a complex interplay between sleep health, physical activity, and body composition. All studies except two [51,55] utilised anthropometry measures such as BMI and waist circumference. While some findings align in associating sufficient sleep with better (healthier) body composition outcomes in multivariable analyses, there are contradictions regarding the impact of factors like sedentary behaviour, noise exposure, and specific health conditions on sleep and cardiometabolic risk.

### Association between disordered sleep and medical interventions

Two studies provided information on the effect of medication on the sleep health of adolescents as shown in Table 3. The studies by Cockcroft et al. in South Africa and Cheikh *et al.* in Tunisia both focused on interventions and their effects on sleep health in specific populations [34,43]. Cockcroft *et al.* conducted a comparative study on children with ADHD, comparing those taking methylphenidate with those not on medication [34]. They found that methylphenidate influenced the timing of sleepiness and perception of sleepiness but did not affect the overall prevalence of sleep disturbance [34]. In their study, significantly higher levels of daytime sleepiness were reported by the parents of the un-medicated children from 13:00 and 15:00 hours when compared to the parents of medicated children, suggesting that treatment of ADHD may result in better sleep of the adolescents and their parents and thus lower daytime sleepiness in the parents/caregivers.

Cheikh et al. studied the effects of melatonin on sleep in adolescent judo competitors by the administering 10 mg of a sustained-release formulation of melatonin after intensive late-evening exercise [43]. He found that the adolescents in the melatonin group had significantly improved total sleep time, sleep efficiency, and other sleep parameters, as well as cognitive performance and subjective sleep quality, compared to the adolescents in the placebo group [43].

Both studies show how medication (methylphenidate and melatonin) can influence sleep-related outcomes in their respective populations. While medication like methylphenidate may influence sleepiness post-administration without affecting the prevalence of sleep disorders, melatonin shows promise in improving various aspects of sleep health in adolescents who have disrupted sleep due to intense physical activity.

## Discussion

### Principal findings

There has been a gradual increase in the number of studies on adolescent sleep in Africa, especially in the past ten years, though the total of 43 papers on adolescent sleep in Africa obtained from the inception of the searched databases reflects overall poor attention to sleep in African adolescents. Though most of these studies were questionnaire-based cross-sectional studies, they provide the baseline information necessary to advance adolescent sleep research in the continent. Across studies, inadequate sleep duration during weekdays (≤ 8 hours) was common among adolescents, often compensated by longer sleep duration on weekends. Additionally, the prevalence of poor sleep quality (ranging from 50.2% [12] to 85% [54]) and the diverse range of sleep disorders reported across various African regions, emphasize the global burden of sleep-related issues in adolescents on the African continent. Sociodemographic variables contributed to the adolescents’ sleep health. Younger age was a protective factor for good sleep health while inconsistencies on the role of sex in adolescent sleep health were noted across regions and may be due to differences in the sleep variables used. The use of electronic devices at bedtime was a major risk factor for sleep disturbances, especially among urban-dwelling adolescents. Also, use of stimulants (khat in Ethiopia, methylphenidate in adolescents with ADHD, and caffeine) was associated with poor sleep health. Furthermore, ACEs and the burden of schoolwork were reported in some studies as risk factors for adolescent sleep disturbances. Poor sleep was associated with poor academic performance, depression, and anxiety, as well as adverse cardiometabolic risk factors. However, the majority of the available studies on sleep and CMR studied only measures of adiposity such as BMI and waist circumference while only one study [51] utilised multiple measures of CMR such as blood pressure, serum glucose, and serum lipids [51]. In contrast, the use of melatonin was reported to improve sleep and performance in adolescents after intense exercise sessions late in the day/close to bedtime.

Among the multi-dimensions of sleep health, sleep duration, quality and timing were the most studied among African adolescents. Sleep duration of adolescents on weekdays was below the recommended range in some questionnaire-based studies [13,15,41], while many demonstrated catch-up sleep on weekend nights [13,15,37,41]. Using objective sleep measures (polysomnography), El-Baroudy *et al* reported a sleep duration of less than 8 hours among children with end-stage renal disease and healthy controls, though their sample size of 40 cases vs 40 controls was small [46]. The ideal sleep duration in adolescents is above 8.5 hours per night [69] with sleep duration >10 hours being reported as long sleep duration. However, only a few studies reported a weekday mean sleep duration at or above the cut-off [39,40] while the weekend mean sleep duration was higher, and the proportion of adolescents with insufficient sleep lower, over the weekend in most studies [39,41]. This pattern is similar to reports for adolescents in different parts of the world [70–72]. Gamble *et al* reported delayed sleep/wake schedules and late weekend wake time, especially among adolescents with a high dose of bedtime electronic device use [72]. The ‘catch-up’ sleep on weekends occurs as a result of the need to pay back the accumulated sleep debt over school days, and it’s often referred to as “social jetlag” [73]. Thus, as in other parts of the world, insufficient sleep is prevalent among African adolescents. A Nigerian study has reported inadequate sleep duration among Nigerian children akin to that observed in other parts of the world [74]. Our recent study has shown that this was particularly true for urban adolescents, who systematically reported lower sleep duration than rural adolescents in Southwest Nigeria [57]. African adolescents are equally affected by insufficient sleep and will require further studies and tailored interventions.

Furthermore, poor sleep quality was reported by more than half of adolescents in all the studies on sleep quality. Some studies reported a prevalence of more than three-quarters of adolescents with poor sleep quality [54]. The reported excessive daytime sleepiness in these studies is mostly a reflection of the poor quantity and quality of nighttime sleep in these adolescents. Aside from daytime sleepiness reflecting inadequate nighttime sleep, it also gives concern about the likelihood of disruptions in daytime functioning among adolescents.

Across diverse settings and populations, several studies reported a high prevalence of sleep disturbances among adolescents. For instance, studies in Cape Verde, Mozambique, Nigeria, South Africa, and Tunisia all highlighted significant percentages of adolescents experiencing sleep issues. The most prevalent sleep problems were insomnia, parasomnias and sleep-related breathing disorders. There were no results on the prevalence of hypersomnia, though the only case report was on Kleine-Levin syndrome which has recurrent episodes of hypersomnia as one of its features [38]. The lack of reports on disorders of hypersomnia like narcolepsy may be due to the non-availability of sleep laboratories and diagnostic facilities for polysomnography, multiple sleep latency tests and cerebrospinal fluid orexin assays for confirming narcolepsy [19]. Various disparities were noted in the pattern of risk and protective factors affecting sleep health. Using the socioecological model, these factors are better considered as individual, family, neighbourhood and school-level factors [75]. As described by Hale et al, sleep health is socially patterned and contributes significantly to overall health through multilevel factors [2].

At the individual level, inadequate sleep hygiene practices were the most reported in this review. Notably, bedtime use of electronic devices impaired sleep health significantly across different African adolescent populations [15,41,44,49,50,53]. The findings from these studies align with previous research on the negative impact of evening exposure to electronic devices on sleep quality and circadian rhythms among adolescents in other climes [72,76,77] In a study among 1,184 Australian adolescents, a dose-response relationship was reported between bedtime use of electronic devices and delayed bedtime as well as lag in bedtime [72]. Human phase response curves [78,79] to light describe how light in the evening even of moderate intensity results in circadian phase delays, also in adolescents [80], in particular when the spectrum of light includes in the blue range [81], such as in electronic devices. Usage of electronic devices vs. reading a printed book at night before bedtime has been associated with later bedtimes. Delayed bedtimes due to electronic device use, in turn, lead to lower sleep duration as adolescents still need to wake up early to go to school. Furthermore, a systematic review involving 49 studies from Western countries identified artificial light exposure at night, excessive screen time and social media use to be associated with problems falling asleep (longer sleep onset latency) and poor sleep quality among early and middle adolescents [82]. Exposure to light from screens, especially in the evening, leads to circadian phase delays [83,84] and to disruptions in the circadian clock and subsequent sleep disturbances [82]. Although there were variations in the types of electronic devices used by adolescents in this review, the association with adverse sleep health outcomes were common to all. Closely related to screen use is the issue of internet addiction and depression. Bhandari et al., reported that poor sleep quality was associated with internet addiction and depression, suggesting a potential interplay between these factors [85]. A systematic review also emphasized the need to establish the connection between internet addiction and sleep problems, further supporting the idea that internet-related behaviour can influence sleep health. For instance, internet addiction has been reported to act as a partial mediator in the relationship between ACEs and sleep disorders, particularly among female [53]. Adolescents with past ACEs may develop psychosocial issues that make them more interested in virtual relationships and compensatory internet-based habits such as gaming. Thus, research collectively suggests that internet addiction and excessive internet use serve as important pathways to poor sleep quality. However, further studies on the duration of electronic device use, the content of screen and internet use and the behavioural factors that potentiate this harmful sleep hygiene practice are needed to develop appropriate interventions.

Another, individual-level factor affecting adolescent sleep health is substance use. Generally, the association between substance use and sleep disturbances has been reported as bidirectional with many substances causing sleep problems and having a sleep disorder or disturbance also contributing to the tendency of abusing substances.[23] The adverse effects of khat use on sleep and overall well-being have been reported among pregnant women [22], adults [86], and college students [21] in Ethiopia. This is further supported by the findings of a qualitative study on the pattern, determinants and effects of substance use among Ethiopian students where a student was quoted as saying “I won’t be able to sleep if I didn’t smoke shisha. I chew khat daily and if I don’t smoke shisha, I won’t be able to sleep”.[87] Hence, there is a need for attention to be given to reducing substance use when developing sleep-promoting interventions for adolescents. Studies are also needed on the contribution of substance use to adolescent sleep health in Africa.

Furthermore, this review identified other individual-level factors such as age and gender that may be associated with adolescent sleep. Younger adolescents had better sleep than older adolescents, which is consistent with the known larger phase delay in older adolescents compared to younger adolescents [88]. There were disparities in the association between gender and sleep health. While some studies reported a lack of difference in sleep health between genders [12,39,49] a few found a longer sleep duration [15,33] and earlier wake times [29,37] in females while another study reported a shorter sleep duration in boys [48]. Hence, there are inconsistencies in the association between gender and adolescent sleep health in the African studies. These inconsistencies may probably be because of physiological factors, diverse cultural practices, and gender roles that may affect sleep patterns [15]. Differences in sample sizes and methods with respect to sampling and data analysis may have contributed to the disparities in findings. Thus, further studies, especially qualitative studies, are needed to explore and understand the role of gender and cultural factors on the adolescents’ sleep health.

Physical activity was another individual-level factor considered as influencing adolescent sleep. In the study by Garnier *et al,* the shorter sleep duration and poorer sleep quality were associated with higher physical activity in the boys. This result was thought to be due to the cultural differences in the nature of physical activity between adolescent boys and girls in Senegal [33]. While the physical activity for the boys involved farm work, cattle rearing and sports and was mainly done in the evening, girls engaged more in house chores throughout the day [33]. Doing intense exercise in the last two hours before bedtime has been associated with worse sleep in adult studies [85], which may explain those findings. Hence, culturally-mediated differences in gender roles may contribute to the type of physical activities African adolescents engage in which may in turn influence their sleep health. These contributions of culture and gender roles to adolescent sleep health will need to be further explored, possibly with qualitative studies. The findings from the two longitudinal studies from South Africa suggest that physical activity may be a protective factor which promotes adolescent sleep. Though Hanson *et al*’s finding suggests that an adolescent with short sleep and high screen time may not necessarily have low physical activity, Micklesfield *et al* with the birth-to-twenty-plus cohort in South Africa, reported an association between higher physical activity, sufficient sleep, and more favourable body composition outcomes, suggesting that improving sleep and physical activity may improve cardiometabolic health of adolescents [48]. Interestingly, a number of studies on adolescent sleep health in Africa involved adolescents engaging in sports. These studies considered the effect of diet / substance use [66], fasting [56,62] and melatonin use [43] on the sports performance of the adolescents and their sleep health. Hence, promoting physical activity in adolescents is a feasible target for improving the sleep and cardiometabolic health of adolescents generally and especially those involved in sports.

Another risk factor for poor sleep in adolescents identified in this review was ACEs, which encompass traumatic events such as emotional abuse, physical abuse and witnessing violence at home or in the community during childhood [53]. As discussed above, the psychosocial issues stemming from ACEs can drive compensatory behaviours, such as internet addiction, that subsequently disrupt sleep. However, relying on a single study to support the argument regarding ACEs and sleep represents a notable limitation of the current scoping review. Therefore, more primary research is urgently needed to fully understand the impact of childhood trauma and adversities on adolescent sleep health across Africa.

There were disparities in the role of family and household in influencing adolescents’ sleep. The finding of a significant impact of maternal education on adolescents’ sleep trajectories reported by Hanson *et al* shows the importance of parental factors, such as their education on adolescent sleep [45]. Previous studies in other regions of the world have investigated parental education as well as parental work schedule, parental sleeping habits, sleep-related beliefs and adolescent-parent relationships. Olorunmoteni *et al*. in Nigeria associated lower social class and public school attendance with longer weekday sleep duration, suggesting that as socioeconomic status improves, sleep patterns in adolescents may become worse [15]. This unexpected association between higher SES and poorer sleep quality may be due to lower parental oversight over bedtimes and/or higher availability of light-emitting devices at night in high SES households. The findings suggest a complex interplay between socioeconomic status, cultural norms, and individual behaviours in influencing sleep health. Moreover, the studies emphasize the importance of considering a holistic approach that integrates socioeconomic factors into interventions aimed at improving sleep health.

The school-related factors shown to have an association with poor sleep health included a perceived high burden of schoolwork and late closing time [26,54]. The social jetlag described earlier could contribute to the development or exacerbation of sleep disorders like insomnia or circadian rhythm disorders [89]. The findings from the studies in this review align with existing research which demonstrated that delaying school start times provided better opportunities for sleep and improved sleep and daytime functioning in adolescents [90]. While research consistently emphasizes the importance of adequate sleep for adolescent well-being, variations in sleep patterns, school schedules, and associated factors exist across different African populations. Hence, interventions targeting school schedules, including school start and end times, in African communities may help improve the sleep health of adolescents.

The association of school schedules with adolescent sleep was further shown by studies showing that poor sleep was associated with poor academic performance. Also, adolescents who had more sleep problems may have an increased tendency to use substances with a negative effect on academic performance, suggesting a vicious cycle of relationship between sleep problems, academic performance and substance use [22,35] This indicates an interrelationship between harmful individual habits, poor sleep and poor academic habits. While some studies emphasize the association between poor sleep and academic performance or learning [35,36,54] others highlight the importance of good sleep quality for better academic outcomes [29]. It is important to further explore factors that jointly affect sleep and academic performance in adolescents as possible targets towards improving the overall well-being of adolescents.

The study by Olorunmoteni *et al* conducted in Nigeria among in-school adolescents aged 10–19 years found a significant association between poor sleep quality and depression severity. This aligns with findings, which highlighted the link between exposure to stressors and negative mental health outcomes such as depression and anxiety disorders [91]. The association between depression and poor sleep has been described as bidirectional such that poor sleep is associated with higher risk of developing depression while depression is associated with higher risk of developing poor sleep. The cross-sectional nature of the Nigerian study makes it impossible to establish the direction of the relationship between sleep and mental health in adolescents. Hence, longitudinal studies are required to further explore the association between sleep patterns and mental health in adolescents, and the impact of insufficient sleep on various mental health issues of African adolescents. Such further studies will help to improve the understanding of the various factors affecting adolescents’ sleep and mental health for developing comprehensive strategies to address mental health issues associated with sleep disorders during this sensitive part of neurodevelopment that constitutes adolescence.

### Strengths and weaknesses

Our study is the first to map available evidence on adolescent sleep in Africa. It contributes significantly to the literature on the global adolescent population by providing a comprehensive overview of the few studies on adolescent sleep in Africa. This scoping review highlights the trend and geographical distribution of these studies, the various aspects of sleep health and sleep disorders that have been studied, the risk and protective factors previously identified as contributing to adolescent sleep health and the association between the adolescent and various health and scholastic outcomes. We utilised a comprehensive search strategy to search five databases with the largest selection of indexed journals from Africa and included all relevant articles from the inception of each database, since there was no previous review of publications on adolescent sleep health in Africa. Our review identified the gaps for improving adolescent sleep in Africa: the need for studies across all African countries, especially from the East and Central African regions which had the least number of studies. This scoping review also highlighted specific gaps in the literature on adolescent sleep in Africa: the need for studies on hypersomnia, circadian disorders, and other forms of sleep disorders that require polysomnography diagnoses (sleep apnoea, restless legs syndrome, periodic limb movement in sleep, narcolepsy, parasomnias); the impact of infectious diseases like malaria, trypanosomiasis, human immunodeficiency virus on adolescent sleep health in Africa; the association between sleep health and chronic health conditions like sickle cell anaemia, chronic kidney diseases and mental health conditions. The fostering of such studies will be helped by the formation of an African sleep network, and the collaboration of sleep researchers for multi-African countries to conduct studies for a better understanding of some unique aspects of sleep health in the African context such as the association of climate change and malaria prevention. We sought peer-reviewed publications in English, French, Spanish, Portuguese, Italian and Arabic languages since the official languages in most African countries are anglophone, francophone, lusophone and Arabic, to obtain as many studies as are available on adolescent sleep health in Africa [26]. We did not consider grey literature, blogs, web pages, books, conference presentations. Also, the quality of the publications and the strength of the evidence from the studies were not analysed in our review. This limitation of our study can be improved upon by a systematic review and meta-analysis to provide data on the strength of synthesized evidence.

### Unanswered questions and future research

Our findings reveal that adolescent sleep health is understudied in Africa despite the previously reported high prevalence of sleep disturbance or disorders. The diversity in methods and instruments used to obtain data on the prevalence and pattern of these sleep health issues may explain the differences in the prevalence and types of sleep disturbances obtained from different studies. Most reviewed studies utilised questionnaires for studying sleep rather than objective measures such as polysomnography and actigraphy. Furthermore, the questionnaires used to measure sleep characteristics were a mix of validated questionnaires and simple stand-alone questions about sleep. As a result, comparisons of findings are difficult. Hence, we recommend the use of validated sleep questionnaires or instruments, after systematically checking those instruments (developed elsewhere in the world) for internal consistency (Cronbach’s alpha) and construct validity (using factor analysis) when used in the African context [92]. Questionnaires refined to better capture the local specificities on adolescent sleep in Africa should be developed (such as incorporating sleep disturbances from mosquitoes, and/or from the use of specific stimulants like khat in Eastern Africa, or cultural habits which may underlie aspects such as gender differences in sleep), to capture more accurately the multi-dimensions of sleep health in the African context. Furthermore, the array of risk and protective factors identified in the studies reviewed, especially the rural-urban disparities in the factors, shows the need to further study the determinants of sleep health in the African context, using, in particular, mixed-methods designs that involve the adolescents themselves. Finally, some areas of determinants of sleep health remain clearly understudied: in particular, we single out those major themes of future research: i) adverse childhood experiences’ role in adolescent sleep health in Africa (only one study was found focusing on this aspect), ii) contribution of specific infectious diseases such as malaria, sleeping sickness and chronic HIV or tuberculosis infections (no study was found) and iii) genetic contribution to sleep health in Africa. In order to institute centres of excellence in adolescent (paediatric) sleep health in Africa, there is a need for more capacities for objective sleep measurements using actigraphy and polysomnography and the establishment of paediatric sleep laboratories for both diagnostic and therapeutic purposes.

## Conclusion

Overall, while there are variations in sleep patterns and disorders among different populations, common themes of inadequate sleep, a pattern of catch-up sleep on weekends, and the impact of socioeconomic factors underscore the need for comprehensive interventions to promote better sleep health among African adolescents. Also, there is a need for collaborative multi-country research with stronger research designs, longitudinal and/or mixed-methods studies and the use of objective sleep measures to provide stronger evidence for adolescent sleep issues in Africa.

## Supporting information

S1 Table Search strategy for databases searched. (DOCX)

S2 Table Preferred Reporting Items for Systematic reviews and Meta-Analyses extension for Scoping Reviews (PRISMA-ScR) checklist. JBI = Joanna Briggs Institute; PRISMA-ScR = Preferred Reporting Items for Systematic reviews and Meta-Analyses extension for Scoping Reviews. * Where *sources of evidence* (see second footnote) are compiled from, such as bibliographic databases, social media platforms, and Web sites. † A more inclusive/heterogeneous term used to account for the different types of evidence or data sources (e.g., quantitative and/or qualitative research, expert opinion, and policy documents) that may be eligible in a scoping review as opposed to only studies. This is not to be confused with *information sources* (see first footnote). ‡ The frameworks by Arksey and O’Malley (6) and Levac and colleagues (7) and the JBI guidance (4, 5) refer to the process of data extraction in a scoping review as data charting*.* § The process of systematically examining research evidence to assess its validity, results, and relevance before using it to inform a decision. This term is used for items 12 and 19 instead of “risk of bias” (which is more applicable to systematic reviews of interventions) to include and acknowledge the various sources of evidence that may be used in a scoping review (e.g., quantitative and/or qualitative research, expert opinion, and policy document). *From:* Tricco AC, Lillie E, Zarin W, O’Brien KK, Colquhoun H, Levac D, et al. PRISMA Extension for Scoping Reviews (PRISMAScR): Checklist and Explanation. Ann Intern Med. 2018;169:467–473. doi: 10.7326/M18-0850.
(DOCX)
