# Supplementary material for: Patterns, determinants and associated outcomes with adolescent sleep health in Africa: A scoping review
Source: PLoS One. 2026 Sep 28;21(9):e0353671. doi: 10.1371/journal.pone.0353671 (PMC13618945; doi:10.1371/journal.pone.0353671)
Supplement: Supplementary file 1 [file pone.0353671.s001.docx]

**S1 Table. Search strategy for databases searched**

| MEDLINE (via PubMed, 08-February-2025) | | |
| --- | --- | --- |
|  | Search strategy | Number of results |
| #1 | Sleep[Mesh] OR "Sleep quality"[Mesh] OR "Circadian rhythm"[Mesh] OR "Sleep hygiene"[Mesh] OR "Sleep duration"[tw] OR "sleep chronotype*"[tw] OR "Bedtime variabilit*"[tw] OR "Sleep pattern*"[tw] OR "Total sleep time"[tw] OR "Sleep efficienc*"[tw] OR "Sleep sufficienc*"[tw] OR "Sleep habit*"[tw] OR sleep[tw] OR "Sleep Deprivation"[Mesh] OR "Sleep Deprivation/complications"[Mesh] OR "Sleep Initiation and Maintenance Disorders"[Mesh] OR "Sleep Wake Disorders"[Mesh] OR "Disorders of Excessive Somnolence"[Mesh] OR Parasomnias[Mesh] OR "Sleep disorder*"[tw] OR "Sleep insufficienc*"[tw] OR "Sleep difficult*"[tw] OR Insomnia[tw] OR Hypersomnia[tw] OR "Excessive daytime sleepiness"[tw] OR "Sleep-related movement disorder*"[tw] OR "Sleep-related breathing disorder*"[tw] OR "Sleep apnoea"[tw] OR "Sleep apnea"[tw] OR "Obstructive sleep apnoea"[tw] OR "Obstructive sleep apnea"[tw] OR "Daytime sleepiness"[tw] OR “sleep deprivation”[tw] | 299,692 |
| #2 | Adolescent[Mesh] OR Youth[tw] OR "Young people"[tw] OR Teenager*[tw] OR adolescent[tw] | 2,243,445 |
| #3 | "Africa, Western"[Mesh] OR "Africa, Southern"[Mesh] OR "Africa south of the Sahara"[MeSH] OR "Africa, Eastern"[Mesh] OR "Africa, central"[MeSH] OR "Africa, southern"[MeSH] OR "africa, eastern"[MeSH] OR "africa, western"[MeSH] OR "Africa, Northern"[Mesh] OR Cameroon[tw] OR "Central African Republic"[tw] OR Chad[tw] OR Congo[tw] OR "Democratic Republic of the Congo"[tw] OR "Equatorial Guinea"[tw] OR Gabon[tw] OR "Sao Tome and Principe"[tw] OR Burundi[tw] OR Djibouti[tw] OR Eritrea[tw] OR Ethiopia[tw] OR Kenya[tw] OR Rwanda[tw] OR Somalia[tw] OR "South Sudan"[tw] OR Sudan[tw] OR Tanzania[tw] OR Uganda[tw] OR Angola[tw] OR Botswana[tw] OR Eswatini[tw] OR Lesotho[tw] OR Malawi[tw] OR Mozambique[tw] OR Namibia[tw] OR "South Africa"[tw] OR Zambia[tw] OR Zimbabwe[tw] OR Benin[tw] OR "Burkina Faso"[tw] OR "Cabo Verde"[tw] OR "Cote d'Ivoire"[tw] OR Gambia[tw] OR Ghana[tw] OR Guinea[tw] OR Guinea-Bissau[tw] OR Liberia[tw] OR Mali[tw] OR Mauritania[tw] OR Niger[tw] OR Nigeria[tw] OR Senegal[tw] OR "Sierra Leone"[tw] OR Togo[tw] OR Algeria[tw] OR Egypt[tw] OR Libya[tw] OR Morocco[tw] OR Tunisia[tw] OR "cape verde"[tw] OR Cameroon[ad] OR "Central African Republic"[ad] OR Chad[ad] OR Congo[ad] OR "Democratic Republic of the Congo"[ad] OR "Equatorial Guinea"[ad] OR Gabon[ad] OR "Sao Tome and Principe"[ad] OR Burundi[ad] OR Djibouti[ad] OR Eritrea[ad] OR Ethiopia[ad] OR Kenya[ad] OR Rwanda[ad] OR Somalia[ad] OR "South Sudan"[ad] OR Sudan[ad] OR Tanzania[ad] OR Uganda[ad] OR Angola[ad] OR Botswana[ad] OR Eswatini[ad] OR Lesotho[ad] OR Malawi[ad] OR Mozambique[ad] OR Namibia[ad] OR "South Africa"[ad] OR Zambia[ad] OR Zimbabwe[ad] OR Benin[ad] OR "Burkina Faso"[ad] OR "Cabo Verde"[ad] OR "Cote d'Ivoire"[ad] OR Gambia[ad] OR Ghana[ad] OR Guinea[ad] OR Guinea-Bissau[ad] OR Liberia[ad] OR Mali[ad] OR Mauritania[ad] OR Niger[ad] OR Nigeria[ad] OR Senegal[ad] OR "Sierra Leone"[ad] OR Togo[ad] OR Algeria[ad] OR Egypt[ad] OR Libya[ad] OR Morocco[ad] OR Tunisia[ad] OR "cape verde"[ad] | 769,228 |
| #4 | #1 AND #2 AND #3 | 639 |

| Scopus (via EBSCOHost, 08-February-2025) | | |
| --- | --- | --- |
|  | Search strategy | Number of results |
| #1 | (TITLE-ABS-KEY (“sleep health” OR “sleep duration” OR “sleep chronotype” OR “Bedtime variability” OR “Sleep pattern” OR “sleep”)) | 332,848 |
| #2 | (TITLE-ABS-KEY (“sleep problems” OR “sleep disorders” OR “sleep deprivation” OR “Sleep Deprivation” OR “Sleep Wake Disorders”)) | 102,269 |
| #3 | (TITLE-ABS-KEY (Adolescent OR youth)) | 2,586,760 |
| #4 | (TITLE-ABS-KEY ( “South Africa” OR “Nigeria” OR “Angola” OR “Benin” OR “Burkina Faso” OR “Burundi” OR “Cameroon” OR “Cameroun” OR “Canary Islands” OR “Cape Verde” OR “Central African Republic” OR “Chad” OR “Comoros” OR “Congo” OR “Democratic Republic of Congo” OR “DR Congo” OR “Cote D’ivoire” OR “Ivory Coast” OR “Kenya” OR “Lesotho” OR “Liberia” OR “Madagascar” OR “Malawi” OR “Mali” OR “Mauritius” OR “Mozambique” OR “Mocambique” OR “Namibia” OR “Niger” OR “Principe” OR “Reunion” OR “Rwanda” OR “Sao Tome” OR “Senegal” OR “Seychelles” OR “Sierra Leone” OR “Somalia” OR “Sudan” OR “Swaziland” OR “Tanzania” OR “Togo” OR “Uganda” OR “Zaire” OR “Zambia” OR “Zimbabwe” OR “South Sudan” OR “Ghana” OR “Ethiopia” OR “Botswana” OR “Gabon” OR “Eritrea” OR “Guinea-Bissau” OR “Equatorial Guinea”)) | 942,880 |
| #5 | #1 AND #2 AND #3 AND #4 | 196 |
